# Comparative Potential of Chitinase and Chitosanase from the Strain *Bacillus thuringiensis* B-387 for the Production of Antifungal Chitosan Oligomers

**DOI:** 10.3390/biotech14020035

**Published:** 2025-05-08

**Authors:** Gleb Aktuganov, Alexander Lobov, Nailya Galimzianova, Elena Gilvanova, Lyudmila Kuzmina, Polina Milman, Alena Ryabova, Alexander Melentiev, Sergey Chetverikov, Sergey Starikov, Sergey Lopatin

**Affiliations:** 1 Ufa Institute of Biology, Ufa Federal Research Center of Russian Academy of Sciences, 69, Prospect Oktyabrya, 450054 Ufa, Russia; galnailya@yandex.ru (N.G.); gelena@anrb.ru (E.G.); ljkuz@anrb.ru (L.K.); millinariya@yandex.ru (P.M.); alenarya@rambler.ru (A.R.); mlnt@anrb.ru (A.M.); che-kov@mail.ru (S.C.); senik0406@gmail.com (S.S.); 2 Ufa Institute of Chemistry, Ufa Federal Research Center of Russian Academy of Sciences, 71, Prospect Oktyabrya, 450054 Ufa, Russia; lobovan@anrb.ru; 3 Institute of Bioengineering of Federal Research Center “Fundamentals of Biotechnology” of Russian Academy of Sciences, 7, bld. 1, 60 let Oktyabrya Prospect, 117312 Moscow, Russia; lopatin@biengi.ac.ru

**Keywords:** chitinase, chitosanase, constitutive enzymes, chitooligosaccharides, low-molecular weight chitosan, *Bacillus thuringiensis*, protein purification, fungicidal activity

## Abstract

The depolymerization of chitosan using chitinolytic enzymes is one of the most promising approaches for the production of bioactive soluble chitooligosaccharides (COS) due to its high specificity, environmental safety, mild reaction conditions, and potential for development. However, the comparative efficacy of bacterial chitinases and chitosanases in terms of yield, solubility, and antimicrobial activity of produced COS remains understudied. In this work, chitinase (73 kDa) and chitosanase (40 kDa) from the strain *Bacillus thuringiensis* B-387 (Bt-387) were purified using various chromatographic techniques and compared by their action on chitosan (DD 85%). The molecular mass and structure of generated COS was determined using TLC, LC-ESI-MS, HP-SEC, and C^13^-NMR techniques. Chitosanase converted the polymer more rapidly to short COS (GlcN_2_-GlcN_4_), than chitinase, and was more specific in its action on mixed bonds between GlcN and GlcNAc. Chitosanase needed a noticeably shorter incubation time and enzyme–substrate ratio than chitinase for production of larger oligomeric molecules (Mw 2.4–66.5 and 15.4–77.7 kDa, respectively) during controlled depolymerization of chitosan. Moreover, chitosanase-generated oligomers demonstrate better solubility and a higher antifungal activity in vitro against the tested plant pathogenic fungi. These features, as well as the high enzyme production and its simplified purification protocol, make chitosanase B-387 more suitable for the production of antifungal chitooligomers than chitinase.

## 1. Introduction

Chitin, a globally widespread natural polymer of animal and fungal origins, is a unique and promising resource for the manufacturing of numerous innovative materials and bioactive compounds in various areas of the economy and the social sphere [1,2]. Chitin consists mainly of N-acetyl-2-amino-2-deoxy-D-glucopyranose residues linked by 1,4-β-glycosidic bonds. It is a structural analog of cellulose, the most abundant structural polysaccharide in the plant kingdom [3,4]. The functionality of native chitin is largely confined due to its crystalline supramolecular structure and its insolubility in aqueous solutions [5]. A significant improvement in functional characteristics of chitin is achieved by means of its alkaline deacetylation resulting in the removal of 70–99% of acetyl groups in acetamide at the C2 atom. Chitosan is a deacetylated, highly functionalized derivative of chitin with augmented spectrum of biological activities and promising physicochemical characteristics that make this biopolymer useful for further modifications [6,7,8]. The key structural feature of chitosan is its polycationic nature attributable to presence of protonated NH_2_-groups in glucosamine (GlcN) units whose content can vary from 50% and more. Thus, the total positive charge of a chitosan molecule is determined by a deacetylation degree (DD). This characteristic, together with the molecular weight (M_w_) of chitosan, crucially influences its biological activity and physicochemical properties, including solubility in aqueous solutions [9]. The primary amino group (C2) and hydroxyl groups (C3, C6) in the GlcN unit are responsible for the high reactivity of chitosan and its ability to undergo various chemical modifications (cross-linking, graft copolymerization, alkylation, sulfonation, etc.) [10].

Naturally occurring chitosan is found mainly in cell walls of *Zygomycota* fungi, but the potential proportions of its production are not comparable with the current commercial manufacturing of this biopolymer from marine crustaceans and other invertebrate animals [6,11]. Despite the good solubility of chitosan in significantly diluted solutions of mineral and organic acids, its applicability is limited by high viscosity and insolubility in aqueous solutions with pH values > 6 [12]. This problem solving is realized using detailed practice of depolymerization based on diverse approaches, including physical, chemical, enzymatic and combined methods [13,14,15]. Partial depolymerization of chitosan allows to produce water-soluble bioactive oligomers (COS) with required molecular-mass characteristics, which could be used as initial materials for further chemical or enzymatic alterations [16,17,18]. Better functionality of COS, compared to the original polymer often manifests itself through alteration and enhancement of its biological and physiological activities due to ability to penetrate into target cells. Apart from antimicrobial and antiviral actions, COS display a highly versatile biological activity, including antioxidant, antitumor, immunomodulatory, anti-inflammatory, hypoholesteremic, hypoglycemic effects, among others. These properties contribute to the high application potential of COS in various fields, primarily in medicine, pharmacology, food industry, and agriculture [19]. If the antibacterial and antifungal activity of COS significantly vary in a wide range of its Mw (0.7–20 kDa or more), then some of the listed activities (antioxidant, antitumor, etc.) are maximal in oligomers with a relatively low polymerization degree (PD, n = 4–10) [20].

Enzymatic preparation of COS is seen as the most appropriate tool in terms of high specificity, environmental safety, mild reaction conditions, low energy consumption, and development potential [21]. To date, chitinases (EC 3.2.1.14; 3.2.1.200; 3.2.1.201) and chitosanases (EC 3.2.1.132) are most extensively studied and effective enzymes in terms of “green” production of functional COS. The principal difference in chitinases and chitosanases is their specific hydrolytic activity toward the 1,4-β-linkages between residues of N-acetyl-D-glucosamine (GlcNAc-GlcNAc) or D-glucosamine (GlcN-GlcN), respectively. At the same time, both enzymes are able to break the mixed types of bonds between GlcNAc and GlcN, which are present in chitosan and, to some extent, in chitin. The hydrolytic efficiency of chitinolytic enzymes depends directly on acetylation degree (DA) and pattern (PA) of the chitosan substrate [22,23]. Chitinases hydrolyze the partially N-acetylated chitosan better, and their activity increases essentially along with DA increment, which was confirmed in the case of ChiA and ChiB from *Serratia marcescens* [23]. Chitosanases hydrolyze chitosan with DD 80–85% more rapidly and deeply than chitinases as well as non-specific enzymes such as papain, cellulase, lysozyme, lipase, and others. This feature is considered disadvantageous for effective production of antimicrobial COS, since the final products of chitosan cleavage by chitosanases include mainly inactive or weakly active lower oligomers—as a rule, (GlcN)_2_-(GlcN)_4_ [24,25,26]. It is a known fact that the antifungal activity of lower COS begins to appear notably from PD ≥ 4 [27]. According to many studies, the inhibition of fungal growth by chitosan oligomers is correlated with its Mw up to a certain limit, reaching maximal values for relatively large molecules, composed of several dozen (20–100) of GlcN units. For example, minimal inhibition concentration of chitosan oligomers against various *Candida* species increases by 1–2 orders for COS with Mw 8.4–20 kDa compared to lower COS with Mw 0.73–2.1 kDa (PD = 4–11) [28]. The similar results were reported for COS with 1, 3, 5, and 10 kDa against a number of molds and phytopathogenic fungi—*Aperfillus fumigatus*, *Botrytis cinerea*, *Fusarium solani*, and others [29]. Several bacterial chitosanases can be effectively used to produce (GlcN)_5_, (GlcN)_6_ and longer-chain COS, which demonstrate higher antimicrobial activity [30,31,32,33,34]. The chitinases, along with the cellulases and other non-specific hydrolases are often considered more suitable for the preparation of large oligomeric molecules such as low-molecular weight chitosan (LMWC) with M_w_ 6–16 kDa or other M_w_ ranges, due to their lower specificity towards chitosan (DD 80–90%) [21,35].

Although the specificity of the most studied bacterial chitinases toward chitosan was studied in sufficient detail at the biochemical and molecular levels [22,23], the comparative efficacy of chitosan hydrolysis by chitinase and chitosanase in terms of the yield, solubility, and antimicrobial activity of produced COS is still understudied. In particular, it relates to bacterial cultures that are capable of simultaneous production of both chitinases and chitosanases. The species *Bacillus thuringiensis*, one of the few explored representatives of soil endospore-forming bacteria, is capable of actively secreting chitinase and chitosanase [36,37,38,39]. Chitinases from *B. thuringiensis* have been sufficiently well studied in terms of their involvement in the bacterial pathogenesis against agricultural pest insects [36,39]. Additionally, these enzymes are often considered as potential antifungal bio-pesticides and catalysts for the production of bioactive oligosaccharides from chitin [39]. In contrast, chitosanases from *B. thuringiensis*, although their biochemical characterization is relatively detail [37,38] remain insufficiently studied in the listed aspects. Thus, chitinases and chitosanases from *B. thuringiensis* represent suitable objects for a comparative study on effectiveness of these enzymes in chitosan depolymerization with the aim of producing antifungal COS.

In this work, chitinase (73 kDa) and chitosanase (40 kDa) produced by the entomopathogenic strain of *Bacillus thuringiensis* B-387 (Bt-387) were isolated and compared in terms of chitosan hydrolysis. This strain is interesting because of its ability to differentially induce chitinases and chitosanases, and it is a promising producer of the chitinolytic enzymes with several potential applications [40]. The efficiency of the chitosan conversion using the purified enzymes was assessed based on the M_w_ and PDI values of the produced COS, its antifungal activity, as well as the enzymes’ expenditure and the culture production potential. The influence of depolymerization reaction time and enzyme–substrate ratio on the COS yield, solubility, and antimicrobial activity was discussed. The probable mechanism specificities of both enzymes were supposed by its reaction products’ structure.

## 2. Materials and Methods

### 2.1. Materials and Reagents

The refined flake chitin from the crab shell manufactured and supplied by OOO BioProgress Co. (Shchelkovo, Russia) was used as original raw material for preparation of colloidal chitin according to the previously reported modified methodology reported by Rodriguez-Kabana et al. (1983) [41]. Deacetylated (DD 85%, M_w_ 370 kDa) and partially N-acetylated (DD 50% M_w_ 200–220 kDa) chitosans were provided courtesy of the Laboratory of Biopolymer Engineering of Institute of Bioengineering of Federal Research Center “Fundamentals of Biotechnology” of the Russian Academy of Sciences (Moscow, Russia).

The sorbents for liquid chromatography such as Phenyl-Sepharose CL 4B and CM-Sepharose Fast Flow were acquired from GE Healthcare (Piscataway, NJ, USA). The monosaccharide and oligosaccharides standards for TLC and HPLC analyses were purchased from Sigma Chemical Co. (St. Louis, MO, USA) and MedChemExpress LLC (Monnmouth Junction, NJ, USA). The molecular weight standards for HP-SEC of LMWC were obtained from PSS (Mainz, Germany).

Glycol–chitin for zymographic assay of chitinase after SDS-PAGE was prepared from commercial glycol–chitosan (Sigma-Aldrich, St. Louis, MO, USA) according to Li et al. (2013) [42]. All reagents used in the work were analytical, high, and chromatography grade, including buffers salts, solvents for TLC, and HPLC, etc.

### 2.2. Methods

#### 2.2.1. Bacterial Strain, Its Maintenance, and Cultivation Media

The entomopathogenic strain, *B. thuringiensis* var. *dendrolimus* B-387 from All-Russian Collection of Microorganisms (VKM, Pushchino—Moscow, Russia) was the main object of the present study [accessed on 15 August 2024 https://www.vkm.ru/Catalogue.htm]. The strain is accessible also in the ATCC collection, with number 19266 [accessed on 15 August 2024 https://www.atcc.org/products/19266], and it is known for its former application in a biocontrol of Siberian silkworm as protection measures for Siberian larch and other coniferous species [40].

The strain was maintained by periodic passages on LB agar medium under 35.5 ± 0.5 °C on the medium containing (g/l): KH_2_PO_4_, 1; K_2_HPO_4_•3H_2_O, 0.5; (NH_4_)_2_HPO_4_, 0.5; MgSO_4_•7H_2_O, 0.2; CaCl_2_, 0.1; peptone, 3; yeast extract, 3; corn steep liquor, 1; colloidal chitin, 5; agar, 16 (pH~6.5). For chitosanase and chitinase production, the strain was cultivated in the standard LB broth and in the medium with colloidal chitin (CCM), respectively. The last one contained (g/L): KH_2_PO_4_, 1.0; K_2_HPO_4_•3H_2_O, 0.5; (NH_4_)_2_HPO_4_, 0.5; MgSO_4_•7H_2_O, 0.20; CaCl_2_, 0.10; peptone, 4.0; yeast extract, 3.0; corn steep liquor, 2.0; and colloidal chitin, 10.0 (by dry weight), pH ~6.7–6.8 before autoclaving. The strain B-387 was preliminary grown in 50 mL of LB broth overnight in 250 mL Erlenmeyer flasks on Innova 40R shaker (New Brunswick Scientific, Edison, NJ, USA) under 36 ± 0.1 °C and 220 rpm. Overnight culture (1.77 ± 0.77 × 10^9^ CFU/mL) was inoculated into LB and CCM media at the ratio 1:50 (*v*/*v*) and grown for 96 h under similar conditions. Hereafter, the liquid culture was clarified from the bacterial biomass and cell debris by centrifugation for 30 min (8000 rpm at 5 °C), and the resulting supernatant was used as an initial crude enzyme preparation for a subsequent purification process.

Bacterial growth in LB broth was determined spectrophotometrically by absorption of the culture at 600 nm (OD_600_) on UV-VIS spectrophotometer SF-56 (LOMO-Spectre, St. Petersburg, Russia). Additionally, the bacterial cell titer in LB and CCM liquid media was estimated using the method of serial dilutions and colony counting on LB agar.

The effect of chitosan (DD 85%) on the production of chitosanase and chitinase by the strain B-387 was studied during its growth in the balanced medium containing the same mineral components reported above for CCM medium, and the all organic nitrogen sources were substituted with 1% (*w*/*v*) tryptone, while the chitosan was added instead of colloidal chitin in concentrations of 0.25–2.0% (*w*/*v*). The 4% chitosan solution (pH 6) aliquots were sterilized individually (20 min autoclaving at 0.5 atm) from other components of the nutrient medium and added to the medium immediately prior to the culture inoculation. The cultivation was carried out according to the parameters described above.

#### 2.2.2. Purification of Chitinase/Chitosanase and Protein Measurement

The general scheme of the purification of both enzymes is shown in Figure S1. Briefly, on the first step the culture supernatant both with chitinase and chitosanase activity was concentrated approximately 10-fold using ultrafiltration on VivaFlow 200 module (Sartorius, Göttingen, Germany) with 30 kDa cutoff. Then, the resulting concentrates depending on predominant enzyme were subjected to affinity sorption onto 1% (*w*/*v*) colloidal chitin or 0.5% (*w*/*v*) colloidal chitosan (DD 85%), as it was described before [43,44]. The chitinase fraction adsorbed on the colloidal chitin was finally purified using hydrophobic chromatography on Phenyl-Sepharose CL 4B. The enzyme was applied on the column (2.5 × 10 cm) with the sorbent equilibrated by 50 mM tris-HCl (pH 7.50), containing 1 M of ammonium sulfate. The column was washed by equilibrating buffer to remove of non-adsorbed proteins and the chitinase was detected further in the fractions eluted by the 0.5–0 M NaCl stepwise gradient (Figure S2). The eluted fractions containing chitinase activity were collected, desalted by ultrafiltration on VivaFlow 200, and concentrated using adsorption concentrator VivaPore 10/20 (Sartorius, Göttingen, Germany).

The final purification of chitosanase was performed using cation exchange chromatography on CM-Sepharose Fast Flow, according to the previously reported scheme [40].

The purified enzymes were characterized by its composition, purification effectiveness, and molecular mass using denaturating electrophoresis in 12.5% polyacrylamide gel containing 0.1% of sodium lauryl sulfate (SDS-PAGE) [45]. The SDS-PAGE was carried in Mini-PROTEAN Tetra Cell (Bio-Rad, Hercules, CA, USA) at V_const_ = 200 V. The proteins in gel after run were stained by colloidal solution of Coomassie Brilliant Blue G-250 in the mixture of phosphoric acid (85%), ethanol (96%), and water (2:20:80, *v*/*v*), including 8% (*w*/*v*) of ammonium sulfate. PageRuler Broad Range Unstained Protein Ladder (Thermo Scientific^TM^, Waltham, MS, USA) was used as standard for molecular mass estimation.

Chitinase activity was visualized in polyacrylamide gel using zymography procedure. Before SDS-PAGE, the loaded mixture of enzyme preparations and sample-buffer excluding reducing agent (β-ME) was not subjected to heat treatment. For the enzyme renaturation gel after electrophoresis was rinsed in deionized water and soaked in 100 mM sodium acetate buffer (pH 5.5) containing 1% (*v*/*v*) Triton X-100 for 3 h at continuous agitation in the cold. Then, the gel was washed several times with 10 mM sodium acetate buffer (pH 5.5), overlaid with 7.5% (*w*/*v*) polyacrylamide gel containing 0.05% (*w*/*v*) of glycol–chitin in 10 mM sodium acetate buffer (pH 5.5) and incubated for 3 h under 37 °C. After incubation, the gel was immersed for 10 min in a 0.02% (*w*/*v*) solution of Calcofluor white M2R dye (Sigma, St. Louis, MO, USA) in 0.2 M Tris-HCl (pH 8.5). When the dye solution was removed, the gel remained in deionized water for 1 h at room temperature. The hydrolytic zones of the substrate hydrolysis were revealed as dark bands using transiluminator Super-Bright 312 nm TFS-26MX.Edge PAD 26MX V1 (Vilber Lormat, Marne-la-Vallee, France).

The protein concentration in the enzyme preparations was determined by the Warburg–Christian method [46] using the NanoDrop Lite photometer (Thermo Scientific^TM^, Waltham, MS, USA).

#### 2.2.3. Enzyme Activity Assay

The enzymatic activities of chitinase and chitosanase were analyzed according to the previously reported protocol [40]. Briefly, the enzymes solutions (1 mL) were incubated (60 min at 50 °C and pH 6) with 0.5 mL of 0.5% (*w*/*v*) suspension of colloidal chitin or colloidal chitosan (DD 85%), respectively. After incubation, the reaction mixtures were centrifuged (10,000 rpm for 4 min), and the concentration of reducing sugars in supernatants was measured using modified Schale’s method [47]. The one unit of enzymatic activity was defined as 1 μM equivalent of GlcNAc or GlcN (for chitinase and chitosanase, respectively) liberated during 1 min in 1 mL of reaction mixture at described conditions.

Depolymerization of chitosan (DD 85%) by purified chitinase and chitosanase was assessed also by the reduction in kinematic viscosity of the substrate’s solution during 0, 1, 5, 10, 20, 40, 50, and 60 min at 50 °C. The assayed enzyme was added in amounts 10–100 μL per 6 mL of 1.3% (*w*/*v*) chitosan solution in 100 mM sodium acetate buffer (pH 5.5). The kinematic viscosity of the reaction mixture was evaluated with the Ostwald viscosimeter (VPZh-3 type) with capillary diameter 0.82 mm (Ecroskhim Co. Ltd., St. Petersburg, Russia). The viscosity value (m^2^/s) was deduced by the formula V = g/9.807 × T × K, where g—acceleration of gravity in measurement point; T—flow time of working solution; and K = 0.03 mm^2^/s^2^—the viscosimeter constant. The negative control for viscosity dynamics measurement included the equivalent volumes of 100 mM sodium acetate (pH 5.5) taken in place of enzyme solution. All measurements were carried out in triplicate.

Proteolytic activity in the batch culture of *B. thuringiensis* B-387 was assayed according to the modified method by Haab et al., 1990 [48]. In short, 0.3 mL of 1% (*w*/*v*) azokazein (Sigma, USA) solution in sodium phosphate (50 mM, pH 6) supplemented with 10 μL β-ME was added to 0.45 mL of the enzyme properly diluted in the same buffer. The reaction was stopped after 30 min of incubation at 50 °C by adding 0.75 mL of 5% (*w*/*v*) trichloroacetic acid (TCA), and the mixture was centrifuged (12,000 rpm, 5 min) to remove the non-hydrolyzed substrate. The absorbance of the resulting supernatant was measured spectrophotometrically at 366 nm (A_366_) relative to the same non-incubated mixture. The enzyme quantity leading to an increase in free dye absorbance by 0.1 U for 1 min in 1 mL of the reaction mixture was defined as one arbitrary unit (AU) of protease activity.

#### 2.2.4. Analysis of Biochemical and Catalytic Properties of the Enzymes

The influence of pH and temperature on the activity and stability of purified chitinase B-387, as well as effect of metal cations, detergents, and other additives on this enzyme behavior was evaluated using the analogous protocols that were reported previously [40,44,49]. The substrate specificity of the enzyme was characterized in terms of its hydrolytic activity toward colloidal chitin, chitosan (DD 85% and 50%), carboxymethyl cellulose sodium salt (CMC-Na) of medium viscosity (“Sigma”, St. Louis, MO, USA), amorphic cellulose Sigmacell type 20 (“Sigma”, St. Louis, MO, USA), β-glucan from barley (“Sigma”, St. Louis, MO, USA), birch xylan (“Sigma”, St. Louis, MO, USA), and galactomannan from locust bean gum (“Sigma”, St. Louis, MO, USA). The enzyme reactions were incubated at standard conditions for the chitinase assay (0.5% of substrate concentration, 60 min at 50 °C and pH 6). The relative activity of chitinase against different substrates was presented as percentage portion of its activity indicated against colloidal chitin (100%). The main kinetic characteristics of chitinase such as Michaelis-Menten constant (K_M_) and maximum velocity (V_max_) were calculated from Lineweaver–Burk linear plot constructed from experimental data according to Equation 1/V = K_M_/(V_max_ × [S]) + 1/V_max_.

#### 2.2.5. TLC- and MS-HPLC-Analyses of Chitin/Chitosan Breakdown Products by Chitinase and Chitosanase

The lower chitooligomers generated by enzymatic hydrolysis of chitin and chitosan were analyzed through the thin-layer chromatography (TLC) on Silica gel 60 F_254_ with aluminum support (10 × 20 cm) (Merck, Darmstadt, Germany). Purified chitinase of Bt-387 (~0.19 U) diluted 10-fold by deionized water (10 + 90 μL) was incubated with equivalent volume (100 μL) of 0.5% chitosan (DD 85%, pH 6) at 50 °C. The aliquots of reaction mixture (20 μL) were sampled after several time intervals, heat-treated for 5 min at 100 °C in water bath, and applied in a volume 1–2 μL on the silica gel sheet. The sheet was placed in a closed TLC chamber (Merck, Germany, 20 × 20 cm) containing the mixture n-BuOH—NH_4_OH (28%)—MeOH—H_2_O (5:4:2:1) as a mobile solution system. After maximal running of the mobile phase, the silica gel sheet was dried, sprayed by ninhydrin solution in acetone (0.25% *w*/*v*), and subjected to staining of migrating COS generated at chitosan hydrolysis. The mixture of GlcN (Sigma, USA) and GlcN_2_-GlcN_6_ (MedChemExpress LLC, Monnmouth Junction, NJ, USA) was used as mobility (R_f_) standards for COS identification. The products of chitosan DD 85% and DD 50% hydrolysis by chitosanase and chitinase B-387, respectively, were analyzed in the same way.

For analysis of chitin degradation products, the mixture (0.25 mL + 0.25 mL) of purified chitinase solution (~0.12 U) in phosphate–citrate buffer (50 mM, pH 6) and 0.5% colloidal chitin suspension was incubated at 50 °C. The reaction mixture was sampled after 0.5, 1, 2, 3, 4, 5, and 24 h of incubation and left for 5 min in boiling water bath for chitinase inactivation. TLC was carried out with the same mobile phase solution. The migrating COS were visualized on the dried silica gel plate by its treatment with a 0.44% solution (*w*/*v*) of benzaldehyde (Merck, Germany) in a mixture of n-BuOH—HCl (conc.)—EtOH (9:1.5:1) and following heating at 120 °C for 3 min. A mixture of standard sugars included N-acetyl-D-glucosamine (GlcNAc), N, N′-diacetyl-β-D-chitobiose (GlcNAc_2_), and N, N′, N″-triacetyl-β-D-chitotriose (GlcNAc_3_) (all—Sigma, USA).

HPLC analysis of the chitosan hydrolysates was performed using LC-20 Prominence chromatograph equipped with refractive index detector RID-20A (Shimadzu, Kyoto, Japan). The analyzed samples were injected in a volume of 5 µL and separated on the Shodex Asahipack NH_2_P-50 2D column (150 × 2 mm, particle size 5 μm) with pre-column Shodex Asahipack NH_2_P-50G 2A (10 × 2 mm) (SHOWA DENKO K.K, Tokyo, Japan) in following acetonitrile/water (*v*/*v*) gradient elution mode: 0–10 min, 75–50%; 10–15 min, 50–75% and 15–20 min, 75%. The fractionated sugars were eluted at a flow rate of 0.2 mL/min and a column thermostat temperature of 40 °C.

MS-HPLC analysis was carried out on a liquid chromatography on the same column with tandem mass spectrometer LCMS-IT-TOF (Shimadzu, Kyoto, Japan) operating using electrospray ionization (ESI) in negative ion mode with the following parameters: high voltage probe: −3.5 kV; nebulizing gas flow: 1.5 L/min; CDL temperature: 100 °C; heat block temperature: 100 °C; drying gas pressure: 110 kPa; TOF detector voltage: 1.57 kV. Mass spectra were recorded in the range from 200 to 2000 *m*/*z*. Trifluoroacetic acid (TFA) solution was used as a standard sample to adjust sensitivity and resolution, and to perform mass number calibration (ion trap and time-of-flight analyzer). The data collection and handling were carried out using LabSolutions LCMS ver. 3.60 software (Shimadzu, Kyoto, Japan).

#### 2.2.6. Partial Depolymerization of Chitosan by Chitinase/Chitosanase and Molecular Mass Characteristics of Low-Molecular Weight Chitosan’s Fractions

**Figure 1:**
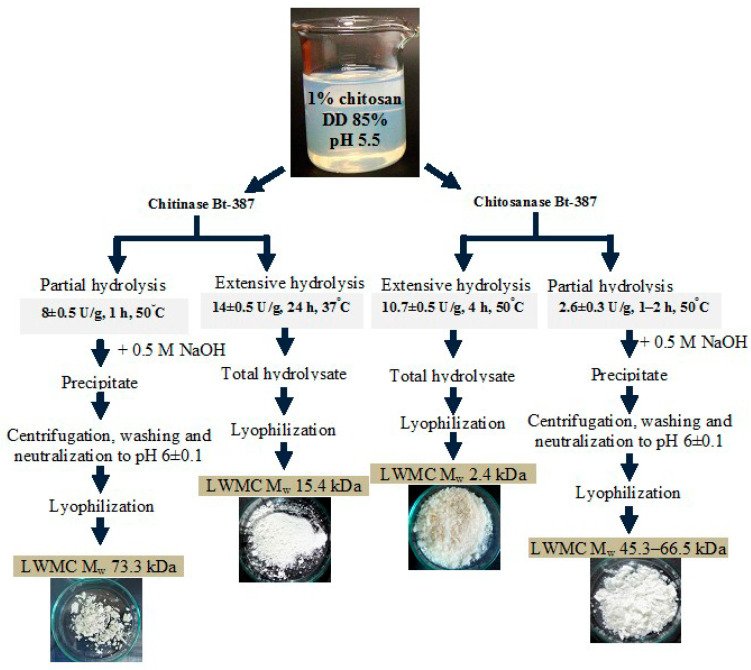
The general scheme of the controlled chitosan (DD 85%) depolymerization by purified chitinase and chitosanase from *B. thuringiensis* B-387.

The limited enzymatic hydrolysis of chitosan (DD 85%) was carried out for 1–2 h at 50 °C after adding of the purified enzyme to a 1% (*w*/*v*) substrate solution at ratio of 1:600 (*v*/*v*). For more extensive hydrolysis, the enzyme–substrate ratio was increased to 1:120 (*v*/*v*), and the incubation time was extended to 4 h (for chitosanase) or 24 h (for chitinase). The hydrolysis degree of the substrate was monitored by the concentration of accumulated reducing sugars determined using the Schale’s method (see above), as well as the measurement of dry weight of residual non-hydrolyzed chitosan. Further the reaction mixture was incubated for 20 min in the boiling water bath for the enzymes’ inactivation. After 1 h enzymatic destruction of the polymer, 4 mL of 0.5 M NaOH was added to the reaction mixture and left for 5 h for complete precipitation of partially hydrolyzed chitosan (LMWC) fractions. Then, the precipitate was collected by centrifugation (30 min at 5000 rpm), neutralized with 0.5 M solution of HCl to pH 6, freeze-dried, and used for further characterization. The dry matter content of the LMWC was determined by weighing (±0.005 g) after its drying at 50 °C to a constant dry weight value. The supernatant was also adjusted to pH 6, freeze-dried, and used as a low-molecular-weight hydrolysis fraction. The mixture of chitooligomers generated by more extensive enzymatic treatment of chitosan (4 and 24 h at enzyme–substrate ratio 1:100) was freeze-dried without the step of precipitation by NaOH. The general scheme of LMWC fractions preparation is presented in Figure 1.

The average molecular weight (M_w_), number-average molecular weight (M_n_) and polydispersity index (M_w_/M_n_) of the prepared LMWC were evaluated using size-exclusion high-performance liquid chromatography (HP-SEC) on the chromatograph S 2100 (Sykam, Eresing, Germany) with degasser K-5004 (Knauer, Berlin, Germany) and refractive index detector RI Detector K-2301 (Knauer, Germany). The separation of LMWCs was performed on the PSS NOWEMA Max analytical 1000 A column (300 × 8 mm, particle size 10 μm) (PSS, Mainz, Germany) using 0.1 M ammonium acetate buffer including 0.2 M NaCl as eluent at pH 4.5, temperature 30 °C and flow rate 1.0 mL/min. The commercial mixture of pullulans with Mw 342, 1260, 6600, 9900, 23,000, 48,800, 113,000, 200,000, 348,000, and 805,000 Da (PSS, Germany) was used as calibration standards. The control and analysis of chromatograms were carried out by means of Multichrom software, v. 1.6 (Ampersand Inc., Moscow, Russia).

The water solubility of prepared chitosan oligomers was determined according to the procedure reported by Kulig et al. (2023) [50] and expressed as limit concentration mg/mL.

#### 2.2.7. NMR Analysis of Chitosan Oligomers’ Structure

The lyophilized samples of LMWCs prepared by partial hydrolysis of chitosan (DD 85%) were dissolved in D_2_O to concentration of 100 mg/mL. The NMR ^1^H, ^13^C and ^15^N spectra were registered on pulsed spectrometer “Bruker” Advance-III (Bruker Corp., Billerika, MS, USA) with working frequency 500.13 MHz (^1^H), 125.47 MHz (^13^C) and 50.58 MHz (^15^N) based on 5 mm PABBO sensor with Z-gradient at constant temperature of the sample 298 K. Two-dimensional spectra were registered in standard modes of multi-pulse sequences of the device software. The commercial samples of D-glucosamine (GlcN) and N-acetyl-D-glucosamine (GlcNAc) (Sigma-Aldrich, St. Louis, MO, USA) were used as standards.

#### 2.2.8. Comparative Antifungal Assay of the Oligomers Generated by Partial Hydrolysis of Chitosan by Chitinase and Chitosanase

The phytopathogenic fungi from the All-Russian Collection of Microorganisms (VKM, Pushchino—Moscow, Russia) affiliated with the World Data Centre of Microorganisms (WDCM) were used as test cultures for in vitro assessment of the antifungal activity of chitosan oligomers. The tested fungal strains included the species *Alternaria alternata* (Fr.) Keisl. F-3047, *Fusarium culmorum* (W.G.Sm.) Sacc. F-844, *F. gibbosum* Appel and Wollenw. F-848, *F. graminearum* Schwabe, 1839 F-1668, *F. oxysporum* Schlecht. emend. Snyder and Hansen F-137, and *F. solani* (Mart.) Sacc. (1881) F-142. Additionally, the causative agent of wheat root rot, *Bipolaris sorokiniana* (Sacc.) Shoemaker IB-G12 available from the microbial collection at the UFA Institute of Biology of UFRC RAS was used as one of the test strains. The previously prepared lyophilized oligomers’ fractions (see Section 2.2.7) were dissolved in sterile MQ-water immediately prior to experiment performance at initial concentration(s) 50–100 mg/mL. The resulting solutions were sterilized using bacterial syringe filters GVS (Sanford, ME, USA) with a pore size of 0.20 μm and used to prepare serial dilutions with sterile MQ-water. The suitable volumes of prepared dilutions were added to 100 μL of test fungal species conidia suspension in sterile potato dextrose broth (PDB) (~10^4^–10^5^ CFU/mL) in 96-well polystyrene cultural plates (Corning Inc., Corning, NY, USA). The medium was titrated previously to pH 5.8 by sterile 1 M solutions of CH_3_COOH or KOH. The final volume was adjusted in the wells to 200 μL with the sterile PDB. The control samples included the same volume of sterile deionized water in place of chitosan oligomers. The conidia suspensions were prepared from the 7 day-old fungal cultures; the cell concentration in the suspension was determined microscopically using the counting chamber. The fungal growth intensity was estimated by the culture optical density at 600 nm (OD_600_) on EnSpier Multimode Plate Reader (Perkin-Elmer, Shelton, CT, USA) after incubation for 72 h at 28 °C. The rate of fungal growth inhibition was determined according to a standard formula: GI = [(K-E)/K] × 100 (%), in which K and E are OD_600_ values in control and experimental variants, respectively.

The effective doses of 50% growth inhibition (ED_50_) of *B. sorokiniana* by several chitosan oligomers were calculated based on the inhibition concentration curves plotted in a range of tested concentrations according to the protocol described above. The minimum inhibition concentration (MIC) was determined as the lowest content of chitooligosaccharides in PDB capable of causing a visible significant reduction in the growth intensity of the tested fungi.

#### 2.2.9. Microscopic Examination of the COS Effect on Fungal Hyphae Morphology

The effect of chitosan oligomers on the growth and morphology of fungal (*B. sorokiniana* and *F. oxysporum*) hyphae and spores in 96-well polystyrene cultural plates was assessed using bright-field microscopy on a Leica DM1000 microscope (Leica Microsystems, Wetzlar, Germany) at magnification to ×400. The visualization and processing of microscopic images was performed using Leica DFC-290 digital camera and LAS 4.12 software (Leica Microsystems, Wetzlar, Germany).

### 2.3. Statistical Analysis

The experiments for the evaluation were carried out in triplicate; the results were expressed as averages X ± SD. The significance of the differences between control and experimental values was estimated by one-way ANOVA (*p* < 0,05) using the ORIGIN PRO 2019b version 9.6.5.169 software (OriginLab Corp., Northampton, MA, USA).

## 3. Results

### 3.1. Bacterial Growth and Chitinase and Chitosanase Production

**Figure 2:**
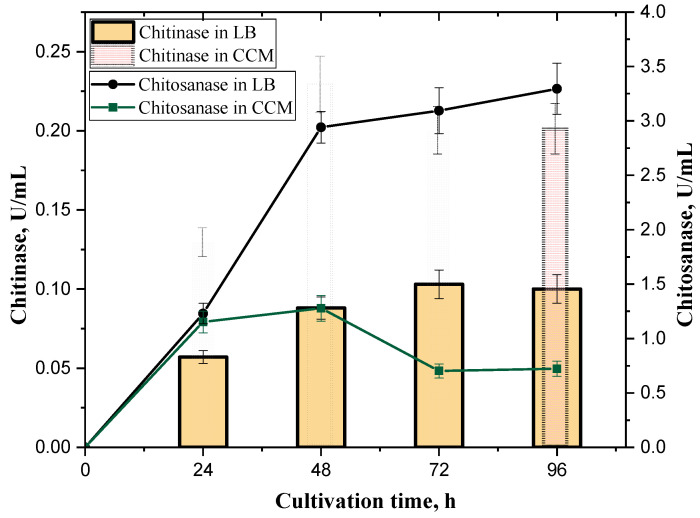
The dynamics of chitinase (columns) and chitosanase (linear graphics) production (U/mL) by the strain *B. thuringiensis* B-387 during its growth in the LB broth (without specific inductors) and medium with 1% (*w*/*v*) of colloidal chitin (CCM) (36 °C, 220 rpm). The confident limits indicate standard error (SE) values.

The strain *B. thuringiensis* B-387 produced actively both chitinase and chitosanase in the presence of colloidal chitin as a substrate during batch cultivation. However, the dynamics of enzymes’ secretion demonstrated the different role of crab shell chitin in induction of these enzymes (Figure 2).

In particular, chitin significantly enhanced the expression of the chitinase in CCM medium, while the production of chitosanase decreased 2–3 times compared to the LB broth. The partial suppression effect of chitin on chitosanase synthesis by *B. thuringiensis* B-387 was observed also in the case of a supplement of 0.4% (*w*/*v*) colloidal chitin into LB broth; however, it was not a significant decline (to 30%) as observed during cultivation in the CCM medium (Figure S3). The growth indices of *B. thuringiensis* B-387 in LB and CCM media were almost identical, except that the bacterial titer dropped more sharply after 72 h in the latter (Table 1). The decrease in bacterial count at least by two orders could be due to significant alkalization of both growth media during the cultivation process (Table 1). This supposition is confirmed by the experimental data demonstrating the growth resumption as well as an increase in total protein and protease production after sterile pH adjusting to 6 after 72 h of cultivation in LB (Figure S4). But this pH balancing did not influence chitinase and chitosanase production levels after 96 h (the data are not presented). The total extracellular protein and protease production by B-387 were higher at its growth in LB (Table 1); that may be due to higher initial content of organic nitrogen in this medium compared to CCM. Nevertheless, higher proteolytic activity here had no essential effect on the stability of both chitinase and chitosanase.

**Figure 3:**
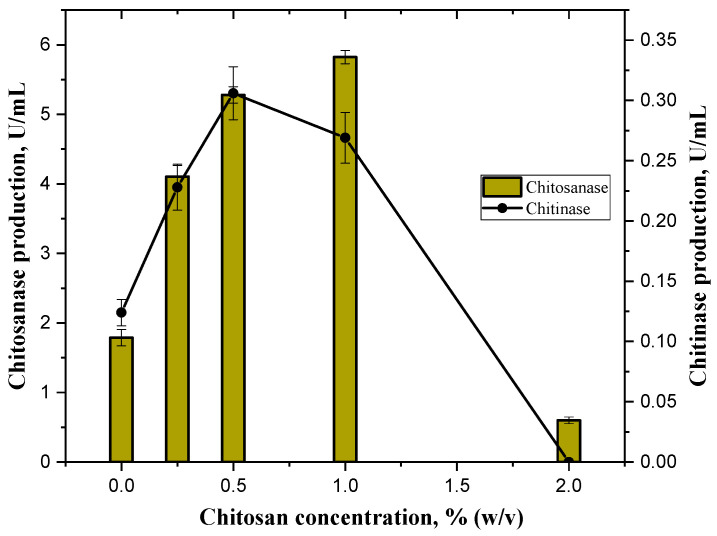
The effect of chitosan (DD 85%) as the main carbon source concentration in the modified LB broth on chitosanase and chitinase production levels by *B. thuringiensis* B-387 after 72 h of cultivation (36^◦^C, 220 rpm). Zero concentration (0) indicates the absence of chitosan in the medium. The confidence limits indicate standard error (SE) values.

Although the strain Bt-387 produced valuable quantities of chitosanase in the LB broth, addition of chitosan (DD 85%) to the cultivation medium resulted in a 2–3-fold increase in the enzyme secretion at substrate concentrations of 0.25–1.00% (*w*/*v*) (Figure 3). Interestingly, chitosan as a carbon source in the absence of the chitin also induced the almost identical augmentation of chitinase production (Figure 3).

The sharp drop in the synthesis of both enzymes at 2% (*w*/*v*) chitosan concentration can be explained most likely by its initial inhibitory effect on the bacterial culture within this threshold range.

### 3.2. Purification Specifics and Main Characteristics of Chitinase and Chitosanase

The purification of chitosanase B-387 was more effective than chitinase at all stages of fractionation, but, at the same time, it resulted in quite a lower yield of the enzyme (Table 2).

**Figure 4:**
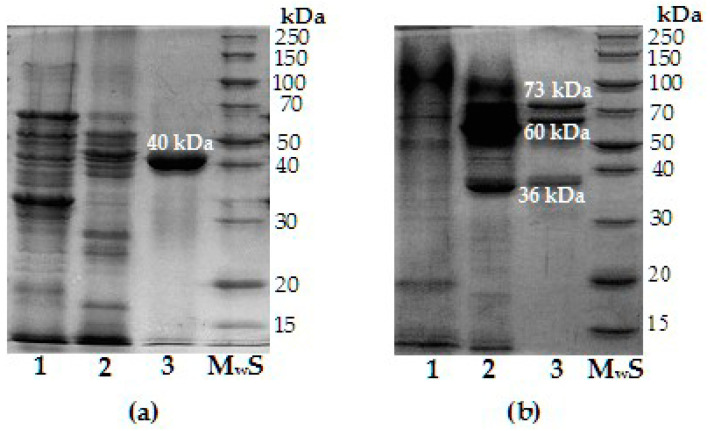
SDS-PAGE of chitosanase (**a**) and chitinase (**b**) preparations after several purification steps from *B. thuringiensis* B-387 grown in LB and CCM media, respectively. Colloidal Coomassie Brilliant Blue G-250 staining. 1—CS; 2—affinity adsorption (on 1% colloidal chitosan and 0.5% colloidal chitin for chitosanase and chitinase, respectively); 3—final purification (CM-Sepharose and Phenyl-Sepharose CL 4B chromatography for chitosanase and chitinase, respectively). M_w_S—molecular weight standard mixture of unstained recombinant proteins PageRuler Broad Range.

SDS-PAGE analysis confirmed these results, demonstrating the homogeneity of chitosanase B-387 after the final purification stage and the presence of several protein bands in the profoundly purified chitinase preparation (Figure 4). Thus, if chitosanase of B-387 was identified as the sole protein with Mw 40 kDa [40], then the purified fraction of chitinase comprised at least three proteins with Mw 73, 60, and 36 kDa (Figure 4b). Chitinase activities of these protein bands were confirmed after their renaturation in undyed gel variant with subsequent cutting of the respective zone and the enzyme extraction. Zymographic assay on a gel containing 0.05% glycol–chitin has also justified the presence of chitinase activity in those protein bands (Figure S5).

In aid of the biochemical characterization, chitinase 73 kDa was additionally purified by re-chromatography on Phenyl-Sepharose CL 4B and collected from the initial eluted fractions of the chitinase peak at low-volume (1–2 mL) fractionation.

### 3.3. Comparative Biochemical and Catalytic Characteristics of Purified Chitinase and Chitosanase

The optimal pH and temperature values for enzyme activity and stability were almost identical both for chitinase (73 kDa) and chitosanase of *B. thuringiensis* B-387 (Table 3). Moreover, both enzymes were similar also in their suppression by various metal cations and detergents, except that chitosanase was significantly more sensitive to zinc cations (Table 3). Among the standard list of tested metal salts, none of the cations showed an activating effect on both enzymes. Unlike chitosanase, the chitinase demonstrated the broader substrate specificity and may slightly degrade substrates such as laminarin, β-glucan, CM-cellulose, and amorphous cellulose (Table S1). Moreover, chitinase quite noticeably hydrolyzed chitosan DD 85% and unexpectedly strongly depolymerized chitosan DD 50% (see below).

**Figure 5:**
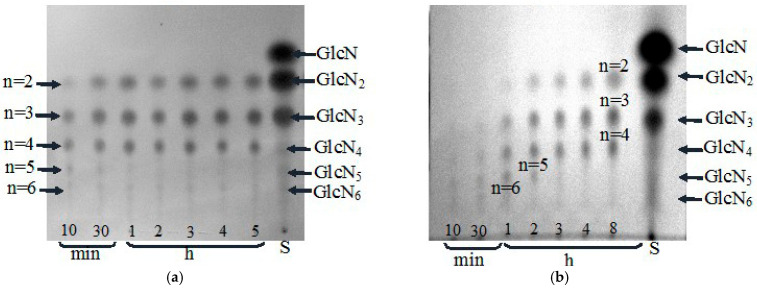
Thin-layer chromatography (TLC) assay on Silica gel 60 F254 (10 × 20 cm) of lower oligomers released during hydrolysis of 0.5% (*w*/*v*) chitosan (DD 85%) by purified chitosanase 40 kDa (**a**) and chitinase 73 kDa (**b**) from *B. thuringiensis* B-387 at standard reaction conditions (50 °C, pH 6). S—commercial mixture of standard chitosan oligosaccharides with defined polymerization degree (n).

**Figure 6:**
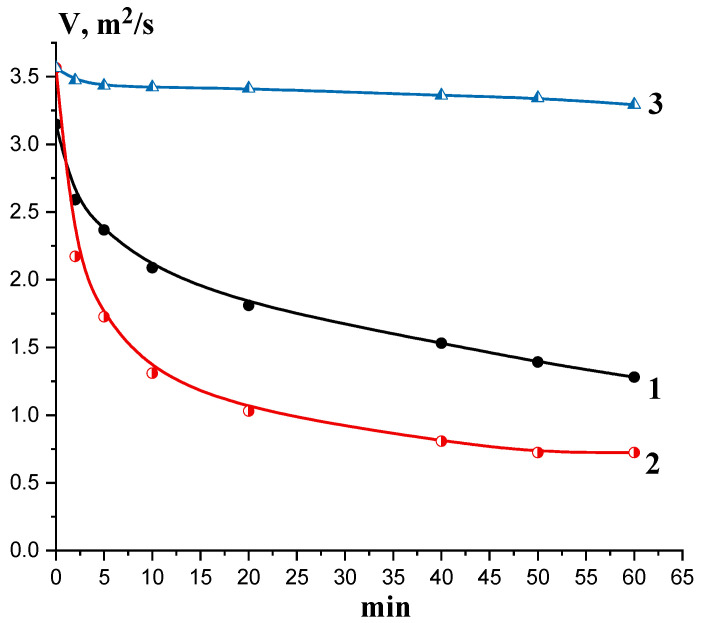
The comparative kinetics of the chitosan (DD 85%) solution (1.3% *w*/*v*) viscosity reduction affected by purified chitinase (1) and chitosanase (2) from *B. thuringiensis* B-387 during 1 h of incubation (50 mM sodium acetate buffer, pH 6, 50 °C). Enzyme/substrate ratio (*v*/*v*): chitinase—1:60; chitosanase—1:120. (3)—control sample (the sample incubated with equivalent volume of distilled water instead of the enzyme solution).

In spite of obvious differences in catalytic activity levels, substrate specificity and kinetic characteristics at hydrolysis of chitosan DD 85%, chitinase (73 kDa), like chitosanase, hydrolyzed this polysaccharide to release chitobiose (GlcN)_2_, chitotriose (GlcN)_3_, and chitotetraose (GlcN)_4_ as main smallest oligosaccharides clearly detected using the TLC procedure (Figure 5a,b). However, as seen in Figure 5, chitosanase showed a more rapid dynamic accumulation of COS with PD = 2–4 and hydrolyzed chitosan more deeply than chitinase, which was also confirmed by viscosimetric assay (Figure 6).

**Figure 7:**
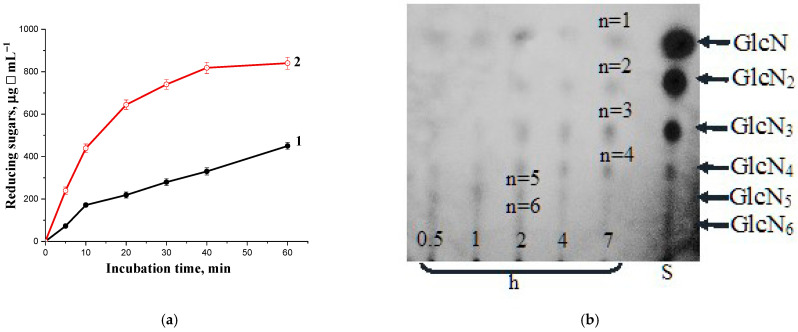
The comparative kinetics of the release of reducing sugars (**a**) at the hydrolysis of colloidal chitin (1) and partially N-acetylated chitosan (DD 50%) (2) by equivalent concentrations of purified chitinase (73 kDa) from *B. thuringiensis* B-387. (**b**) TLC assay of the small COS accumulated during chitosan (DD 50%) degradation by chitinase (73 kDa). S—commercial mixture of standard chitosan oligosaccharides (GlcN2-GlcN6) and D-glucosamine (GlcN). n—polymerization degree of the released COS.

Interestingly, chitinase 70 kDa appears to have slight specificity in the cleavage of mixed types of linkages, such as GlcNAc-GlcN and GlcN-GlcNAc, because it hydrolyzed N-acetylated chitosan (DD 50%) much more rapidly (>2-fold) compared to its specific substrate colloidal chitin (Figure 7a). TLC assay of the 2 h breakdown products of chitosan DD 50% demonstrated the accumulation of the oligosaccharides with a wide range of polymerization degree (n = 1–7), while after 7 h of incubation, the monomer, dimer, trimer, and tetramer predominated in the reaction mixture (Figure 7b).

### 3.4. HPLC-MS Analysis of the COS Generated During Extensive Hydrolysis of Chitosan by Chitinase and Chitosanase

The LC-MS-analysis detected the significant differences in compositions of oligomers released by chitinase and chitosanase of *B. thuringiensis* B-387 during chitosan (DD 85%) hydrolysis, which were not obvious at TLC assay. In the case of chitosanase, according to HPLC-MS data, the COSs mixture was limited by (GlcN)_2_, (GlcN)_3_, and (GlcN)_4_ as major products, similarly with the TLC data—the hydrolytic products of the chitinase action expectedly comprised the significantly broader number of oligomers, where (GlcN)_5_, (GlcN)_6_, and (GlcN)_7_ were dominated based on the relative signal intensity (Table 4).

As distinct from chitosanase, chitinase generated multiple forms of COS, beginning from trimer and ending with nonamer, which contain a single terminal GlcNAc residue (Table 4). However, the deacetylated COS significantly predominated in percent content (~87%), indicating that chitinase is capable of hydrolyzing both types of mixed couplings: GlcNAc-GlcN and GlcN-GlcNAc.

LC-MS assay of the mixture of COS released at partially acetylated (DD 50%) chitosan hydrolysis by chitinase confirmed the TLC data to some extent; in particular, the monomeric molecules comprising both GlcNAc and GlcN were found in significant quantities (Table 4). Generally, acetylated COS containing single GlcNAc residue per its molecule, especially dimer and pentamer, dominated here. The enzymatic production of fully deacetylated COS with n = 2–5 indicates the presence of respective regions in the polymer molecule, suggesting enough irregular distribution of acetylated groups in chitosan with DD 50%.

**Figure 8:**
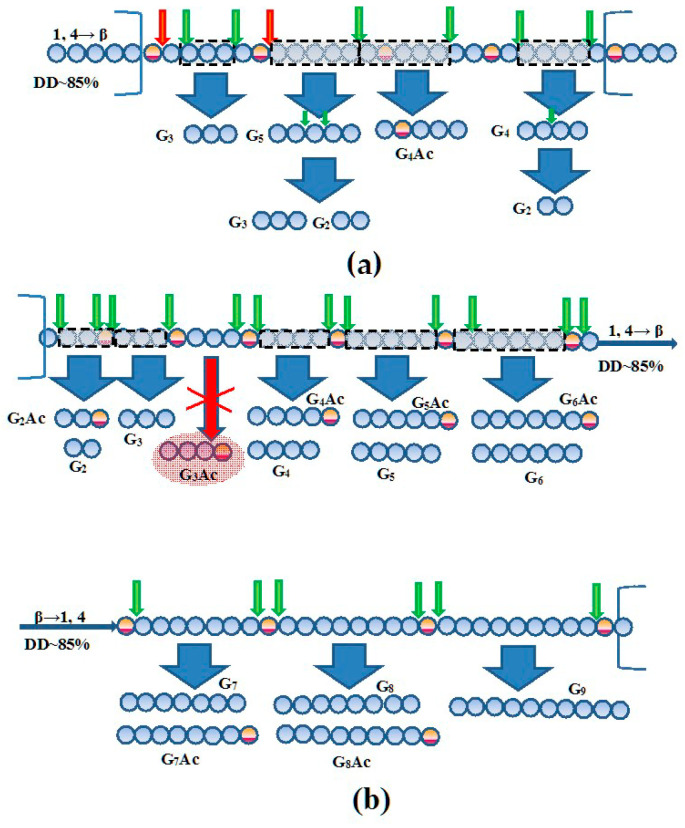
The hypothetical scheme of chitosan (~DD 85%) hydrolysis by chitosanase (**a**) and chitinase (**b**) from *B. thuringiensis* B-387 in view of their possible acetylation patterns. Among the main generated oligomeric products, G1–G9 designate oligomers of GlcN with n = 2–9 and G_n_Ac—oligomers of GlcN containing a single GlcNAc residue. The bluish circles indicate GlcN residues, and two-colored circles—GlcNAc residues. The green arrows indicate obviously preferable bonds for hydrolysis by the enzymes; the red arrows indicate additionally hydrolyzed (in case of chitosanase) mixed bonds. The crossed out large red arrow shows a probable production of tetramer with single acetated residue that was not detected among the products of chitinase.

The probable regions of hydrolysis by chitosanase 40 kDa and chitinase 73 kDa in chitosan DD 85% and chitinase 73 kDa in chitosan DD 50% are illustrated in Figure 8 and Figure S5, respectively, in accordance with the main produced oligomers detected by the ESI-MS assay.

### 3.5. Molecular-Mass Characteristics, Solubility, and Antifungal Potential of the Oligomers Generated at Partial Depolymerization of Chitosan by Chitinase and Chitosanase

Chitosanase hydrolyzed the polymer more effectively than chitinase, as evidenced by the indices of the treatment duration, solubility, and relative antifungal activity (as the ED_50_ concentration values) of the resulting LMWCs (Table 5).

Moreover, the use of chitosanase had the advantage in regard to lower enzyme expenses both in values of the enzyme-substrate ratio and total consumption of the enzyme preparation (Figure 1). With time-limited incubation (1 h) at 50 °C and minor enzyme-substrate ratio (2–2.5 mU/mg), the purified chitosanase Bt-387 converted the substrate to relatively large oligomeric molecules (M_w_~66.5 kDa) without detectable amounts of lower COSs (n < 10) (Figure S7a). Prolongation of the hydrolysis time to 2 h for the same parameters resulted in the formation of smaller LMWCs (Mw~45.3 kDa) with better solubility and lower antifungal activity (Table 5). The yield of this fraction also noticeably declined.

Due to the presence of significant amounts of Cl^−^ and Na^+^ ions falling into the initial reaction mixture during the substrate dissolution, further product precipitation and neutralization, the resulting LMWCs were presented as a salt form with high solubility values in water (Table 5). Finally, a 5-fold increase the enzyme–substrate ratio (to ~10–12 mU/mg) along with extension of the incubation time to 4 h contributed to deep hydrolysis of chitosan, where the lower COSs with weight-average M_w_~2.4 kDa were predominated in the reaction mixture while the alkali-precipitable large oligomeric molecules were not found. TLC assay of this hydrolysate demonstrated the well-detectable amounts of COSs (n ≥ 3–7) with a small admixture of chitobiose (Figure S7b).

Chitinase hydrolyzed chitosan more slowly, in particular 24 h after incubation—a significant enzyme concentration did not result in complete hydrolysis of larger LMWC molecules as in the case of chitosanase (Table 5).

### 3.6. NMR Spectral Analysis of the Partially Hydrolyzed Chitosan Oligomers Produced by Chitinase and Chitosanase

**Figure 9:**
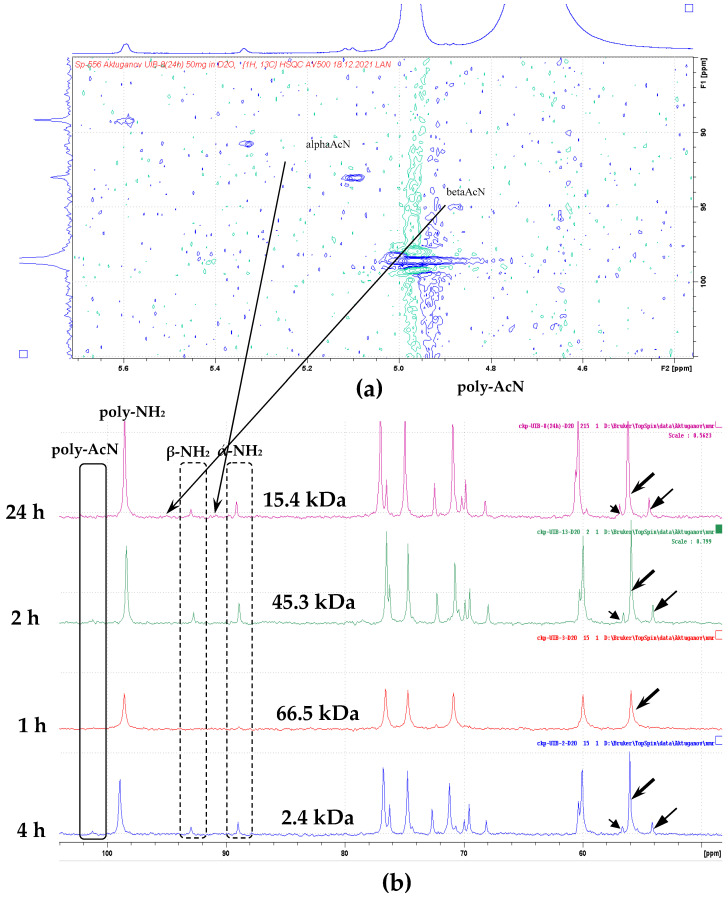
The structural characterization of chitosan oligomers generated after enzymatic hydrolysis of the initial polymer (M_w_ 370 kDa, DD 85%) using NMR spectroscopy. (**a**). Two-dimensional NMR spectrum (^1^H and ^13^C) of LMWC fraction 15.4 kDa produced by purified chitinase of *B. thuringiensis* B-387 (24 h incubation); (**b**) comparative ^13^C-NMR spectra of the same fraction (upper picture in B) and oligomers resulted after 1, 2, and 4 h depolymerization of chitosan by purified chitosanase of *B. thuringiensis* B-387. The short thin arrows indicate the signals of CH_2_-(C_6_) (α + β) (54 and 57 ppm) corresponding to the terminal GlcN and GlcNAc residues. The long thin arrows indicate the signals C_1_ (α + β) (~90 and 93 ppm) corresponding to the terminal GlcN and GlcNAc residues (the regions highlighted by a dashed zone). The medium thick arrows indicate the CH_2_ (C_6_) signal of the inner GlcNAc residues at 56 ppm.

The comparative characterization of the ^13^C-NMR spectra of LMWC resulting after partial (1–2 h) and deep (4 h) hydrolysis of chitosan (M_w_ 370 kDa; DD 85%) by chitosanase B-387 confirmed the higher molecular weight structure of the partially degraded fractions. The terminal GlcN and GlcNAc residues corresponded to the signals C_1_ (α + β) (~90 and 93 ppm) and CH_2_-(C_6_) (α + β) (~54 and 57 ppm), respectively (Figure 9a,b). The mid (inner) residues were evaluated by the C_1_ signals at 99 ppm and CH_2_ (C_6_) at 56 ppm. The major differences between the spectra of the LMWC of 45.3–66.5 kDa and COSs of 2.4 kDa appeared only in the ratio of the intensities of the terminal groups to the mid ones without changing the position of the signals. On the side of the anomeric residue only deacetylated fragments were detected, since the chemical shifts in CH (C_1_) and CH (C_2_) in these are identical to the chemical shift in model GlcN in the complete absence of signals corresponding to the N-acetylated residue. The C^13^-NMR spectra of the hydrolytic products detected exclusively N-acetylated residues as intra-ester (non-hydrolysable) signals for chitosanase action, and the deacetylated ones—for chitinase, confirming the specific action of the both enzymes toward chitosan (Figure 9).

The ratio of anomeric products of chitosan hydrolysis was determined in accordance with the intensity of H^1^-α and H^1^-β signals in the ^1^H-NMR spectra of the oligomer samples.

According to calculations, the ratios of α/β-anomers were about 2:1 and 1.6:1 for chitooligosaccharides generated by chitosanase and chitinase, respectively. This fact evidenced the inverting catalytic mechanism of chitosanase Bt-B387, which is characteristic both of GH 8 and GH 46 chitosanases families. The confirmation for this is commonly found through assessment of the dynamics of changes in reducing groups H^1^-α signals (data are not presented).

### 3.7. Antifungal Effect of Oligomers Generated by Chitosan Hydrolysis Using Chitosanase and Chitinase

In general, the oligomeric products of chitosan (DD 85%) hydrolysis by chitosanase 40 kDa displayed stronger growth-inhibiting activity in vitro against the most of tested phytopathogenic fungi than chitinase-generated oligomers (Table 6). So, the large oligomeric molecules (NaOH-precipitated fraction, M_w_ 71.7 kDa) prepared at the partial hydrolysis of polymer by the chitinase 73 kDa suppressed the growth of fungal strains at significantly higher MIC values than the LMWC fraction (M_w_ 45.4 kDa) produced by chitosanase action (Table 6).

Notably, the more deeply hydrolyzedoligomeric fractions produced by chitosanase and chitinase showed approximately similar MIC values, despite a significant difference in average molecular weights (2.4 and 15.4 kDa, respectively). These fractions were obviously less active in inhibiting fungal growth compared to higher oligomer molecules (M_w_ 66.5, 45.3 and 71.7 kDa) generated by the same enzymes at a limited hydrolysis reaction (Table 5 and Table 6)

The comparative assessment of fungicidal effect exerted by chitosan oligomers towards several fungal species in microplates showed a generally higher sensitivity of the representatives of genus *Fusarium* to its action (MIC~0.01–0.02 mg/mL for chitosanase products, M_w_ 45.3 kDa and MIC~0.04–0.09 mg/mL for chitinase products, M_w_ 71.7 kDa) compared to dark-colored micromycetes such as *A. alternata* and *B. sorokiniana* (MIC 0.20–0.24 mg/mL and MIC 0.31–0.32 mg/mL for chitosanase and chitinase products, respectively; Table 6). A similar consistent pattern was recorded for the deeper-hydrolyzed fractions produced by chitosanase and chitinase (M_w_ 2.4 and 15.4 kDa, respectively), although in its case, the difference between the MIC values toward *Fusarium* spp. and melanin-produced fungal strains was not so significant (Table 6).

**Figure 10:**
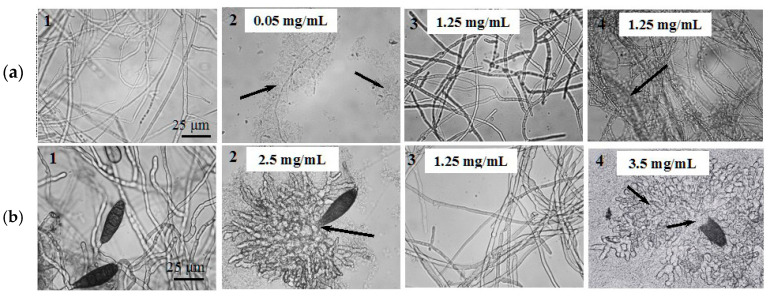
The effects of chitosan (DD 85%) oligomers on the growth morphology of *F. oxysporum* VKM F-137 (**a**) and *B. sorokiniana* IB G-12 (**b**) in potato–dextrose broth (PDB) after 3 days of cultivation in microplates (28 °C). Light microscopy, scale bar = 25 μm. (**a1**,**b1**)—controls. (**a2**,**b2**)—action of LMWC (Mw 45.3 kDa) at different concentrations (partially hydrolyzed product of chitosan by chitosanase B-387); (**a3**,**b3**)—action of COSs (Mw 2.4 kDa) (extensively hydrolyzed product of chitosan by chitosanase); (**a4**,**b4**)—action of LMWCs of 71.7 and 15.4 kDa, respectively, produced by partial and extensive hydrolysis of chitosan by chitinase B-387.

The data of the antifungal assays of chitosan oligomers in microplates were generally supported by light microscopy observations (Figure 10) and the analogous experiments on potato–dextrose agar (PDA) plates.

So, the LMWC fraction M_w_ 45.3 kDa rendered a most pronounced effect on the mycelium development and morphology of *F. oxysporum* F-137 causing a visible lysis of fungal hyphae already at 0.05 mg/mL (Figure 10(a2)), while in the presence of lower oligomers (M_w_ 2.4 kDa), the mycelium morphology remained intact at the concentrations of more than 1 mg/mL (Figure 10(a3,b3)). In relation to *B. sorokiniana* G-12, the visible effect of LWMC fraction M_w_ 45.3 kDa was noted at the higher concentrations (0.4–2.5 mg/mL) and manifested in disruption of the hyphae development, its deformation, vacuolization, and thickening, as well as multiple branching of growth tubes during conidia germination (Figure 10(b2)). The similar effect was caused by the chitinase-generated LMWC fraction M_w_ 15.4 kDa but at the more substantial concentrations (Figure 10(b4)).

## 4. Discussion

The strain *B. thuringiensis* B-387 is capable to produce simultaneously chitinase and chitosanase, which is common for certain groups of the chitinolytic bacteria. A specific feature of Bt B-387 is its ability to secrete significant quantities (3–5 U/mL) of chitosanase in the absence of chitosan as a substrate inductor, while its chitinase complex is synthesized commonly at a 10-fold lower level and requires the supplement of colloidal chitin into the nutrient media. At the first sight, the synthesis of chitosanase by B-387 is constitutive, in contrast to inducible chitinase. This is supported by the fact of much higher chitosanase activity in LB broth compared to chitinase. However, the chitinase production increased by less than 2-fold in the presence of 1% (*w*/*v*) colloidal chitin (CCM medium) compared with its initial level in LB, while chitosan (DD 85%) as specific substrate also induced 2–3-fold increment of chitosanase secretion by *B. thuringiensis* B-387 at concentrations 0.25–0.50% (Figure 3). This fact testifies on nearly similar induction’s levels of chitosanase and chitinase in B-387 by specific substrates, except for much higher basal secretion of chitosanase that indicates its special function. The physiological features of bacteria from the *B. thuringiensis* group often associated with their entomopathogenic behavior implicating the constitutive synthesis of chitinolytic enzymes along with other pathogenicity factors [39]. However, it does not explain the reasons for the decrease in chitosanase production by Bt-387 when colloidal chitin is used as a main carbon source (Figure 2 and Figure S3). As it was found in our unpublished experiments, the depressive influence of chitin on chitosanase synthesis may be smoothed over or enhanced depending on the nature and concentration of the organic nitrogen source as well as on other parameters, including Mg^2+^ content. For example, the negative effect of colloidal chitin (0.25–2% *w*/*v*) on chitosanase production by B-387 was not manifested when lactalbumin hydrolysate or tryptone (1% each) were used as nitrogen sources (the data are not presented). Apparently, the carbon–nitrogen ratio plays a significant role in the level of chitosanase expression in this strain in the presence of exogenous chitin. This is not excluding its similar regulation to that of chitinase(s) at the transcriptional level [39]. The induction of chitosanase by chitin substrates was reported previously for the various bacteria [51]. However, the descending effect of colloidal chitin on chitosanase production under its concurrent expression with chitinase has been recorded for the first time in our research. As to the biotechnological potential of both enzymes, chitosanase from B-387 is more promising in terms of the production level and flexibility in optimizing approaches that are favorable for increasing enzyme yields. In addition, although the high expression of both enzymes requires the addition of an inductor substrate to the growth medium, as reported in most studies [51,52,53,54], the synthesis of chitosanase by B-387 depends less on the presence of chitosan, and it can be significantly increased without the use of this expensive polymer.

The general scheme presented in Figure S1 evidently demonstrates the need for different chromatographic techniques at the final (specific) step of chitinase and chitosanase purification. However, chitinase presents a more challenging problem for isolation of homogenic individual enzymes using traditional approaches and sometimes requires highly laborious fractionation methodology. This fact aligns with the studies of bacterial and fungal chitinases, and it may be explained, as a rule, by more diverse and multiple composition of chitinase complex compared to chitosanase [53,55,56,57,58]. The enzymatic complex of chitinolytic microorganisms comprises commonly chitinases encoded by alone or several genes; some of these are similar in molecular mass, charge, and other properties, what complicates their effective separation.

Chitosanases with molecular masses of about 40 kDa or close the that value, belonging as a rule to the GH8 family, are not rare among various bacilli, including *B. thuringiensis* [37,38,59,60,61,62]. At the same time, most of chitinases characterized as members of *B. thuringiensis* group have a molecular mass of about 70 kDa, although there are also chitinases with Mw~30 kDa [36,39], which is quite consistent with our results. Additionally, as shown by further modifications of the purification scheme, the isolation of chitosanase in a homogenous state did not require the obligatory use of affinity adsorption on colloidal chitosan (the data are not presented), which significantly simplifies and accelerates the preparation of highly purified enzyme. Hence, in view of the purification specifics, chitosanase *B. thuringiensis* B-387 is more suitable than its chitinase(s) for biotechnological application focusing on chitosan depolymerization. The main advantages of chitosanase in terms of purification are easy and reproducible isolation process, remarkable homogeneity, and high specific activity of the final preparation.

As found in biochemical characterization, purified chitinase and chitosanase were quite similar in their main physical–chemical properties. However, they were obviously distinguished by kinetic characteristics and specificity. Chitinase 73 kDa hydrolyzed notably chitosan DD 85% and indicated the minor hydrolyzing action towards CMC, amorphous cellulose, laminarin, and β-glucan (Table S1). The ability to hydrolyze chitosan is an essential feature in most of microbial chitinases [22,23,32,35], while their hydrolytic activity toward the cellulose, laminarin, xylan, and other glucans is not often found [63]. The archaeal chitinase from *Thermococcus kodakarensis* KOD1 is one of unique exclusions among chitinases in terms of its relatively high cellulase activity due to the cellulose-binding domains in its structure [64]. Apparently, the presence of additional carbohydrate-binding modules (CBM) or any variations in the structure of the chitin-binding domain of bacterial chitinases determine their substrate specificity to cellulose and other glucans [65,66]. But the activity of the chitinase Bt-387 (73 kDa) toward the cellulose and other mentioned glucans was close to trace level, that excludes its significant interaction with these polymeric substrates. As distinct from the chitinase, chitosanase from *B. thuringiensis* B-387 did not degrade colloidal chitin, and the velocity of hydrolysis of partially N-acetylated chitosan (DD 50%) by this enzyme decreased approximately 3.4-fold compared to chitosan DD 85% [40]. This fact evidences the high specificity of chitosanase for recognition of GlcN and GlcNAc residues in the substrate molecule. The smallest MS-detectable oligomer comprising single GlcNAc residue among the products released by chitosanase (40 kDa) was pentamer generated in minor quantities (less than 1%). The dominant content (>50%) of chitotriose in the main products of chitosanase may be related to the preferential specificity of the enzyme to GlcN–GlcN bonds. By this route, chitosanase readily cuts (GlcN)_3_ fragment between the neighboring GlcNAc residues repeating approximately every 5–6 GlcN residues, assuming that they are relatively uniformly distributed in chitosan DD 85% molecule (Figure 8a). At the same time, TLC data demonstrate the release of (GlcN)_5_ and (GlcN)_6_ as the minor intermediates at the initial stages of enzymatic reaction, which are rapidly converted to (GlcN)_2-4_ during incubation (Figure 5a). Although, this action mode is typical for the several GH8 family chitosanases, in particular, for chitosanase from *Bacillus* sp. MN [67], probably, chitotriose is an initial non-degradable oligomeric molecule generated at once by chitosanase B-387, while (GlcN)_2_ is generated rather due to secondary hydrolysis of (GlcN)_4_ and the larger COS molecules. The action of chitosanase on N-acetylated chitosan often results to accumulation of oligomers with inner non-hydrolyzed GlcNAc residues [22], and evidently, that the minor acetylated pentamer released by chitosanase Bt-387 contains single GlcNAc at the second position from non-reducing end (Figure 8a), which was confirmed indirectly with the data of NMR-spectroscopy analysis (see Section 3.6).

According to LC-MS results, chitinase (73 kDa) converted chitosan (DD 85%) to the broader number of the oligomers containing a single terminal GlcNAc residue, with DP = 3, 5, 6, 7, and 9 (Table 4). At the same time, the fully deacetylated COS (fdCOS) constituted the overwhelming majority of the products based on the total signal intensity calculations. Among these, the pentamer and hexamer were the most abundant products of chitinase supporting the model of ordered distribution of GlcNAc residues in the substrate molecule (Figure 8b). Nevertheless, enough high concentrations of COS with DP = 2, 3, 4, 7, and 8 indicate that really this distribution may noticeably vary in certain regions of chitosan. As known, microbial chitinases and some non-specific hydrolases degrade chitosan with DD 75–90% to longer-chain COS [19,31,34], since their hydrolytic action is constricted to GlcNAc residues, which are rarely distributed in substrate molecule(s). The high content of fdCOS among the products of chitinase evidence indirectly its ability to hydrolyze both types of mixed couplings GlcNAc–GlcN and GlcN–GlcNAc.

At hydrolysis of partially N-acetylated chitosan (DD 50%) by chitinase, the paCOS containing a single acetylated residue per molecule dominated in the hydrolysate, especially, dimer, trimer, and pentamer (Table 4). Nevertheless, fdCOS are also detected here in notable quantities, supposing the multi-point enzyme–substrate interaction, including the splitting of linkages between both internal and terminal residues, which additionally increases the chitinase efficiency. The found monomers GlcN and GlcNAc could be the secondary products accumulating due to a large number of mixed linkages in the substrate, which are easily hydrolyzed by chitinase (Figure S5). Moreover, diacetylchiobiose was detected among the hydrolysis products, indicating the presence of coupled (GlcNAc)_2_ fragments in the chitosan structure. These fragments can be cleaved from the polymer chains termini by chitinase, similarly to its action on chitin.

Thus, from the viewpoint of preparation of soluble bioactive COSs with n ≤ 10 the chitinase from *B. thuringiensis* B-387 can be considered a more promising enzyme, than chitosanase, which produces COSs with n = 2–4 as major products after extensive polymer hydrolysis. Furthermore, chitinase is more suitable for production of paCOS from N-acetylated forms of chitosan, because it more actively converts such substrates by reason of its broad specificity to all types of mixed linkages. However, the efficiency of chitinase Bt-387 remains questionable in terms of COS total yield, when the highly deacetylated (DD ≥ 80%) chitosan is used as the initial substrate. At the same time, the activity of chitosanase prone to control through the crucial parameters such as the time incubation, enzyme–substrate ratio and temperature, which allow for significant increases in the production of long-chain COSs with n ≥ 5 [22,25,31,32,68,69,70].

Apart from production of defined high-purity COS, suitable for use in medicine and pharmacology, chitosan hydrolysis is often intended to prepare larger soluble molecules (PD > 20) with a more complex and difficult-to-identify composition [21,71,72,73,74].

Compared to short-chain COSs (n ≤ 10) the advantages of such oligomers, which are termed commonly as low-molecular weight chitosans (LMWC) or oligochitosans, is higher biological, including antimicrobial, activities along with improved solubility than in high- and medium-molecular-weight chitosan [71,72,73]. In some limits, LMWC demonstrate higher antibacterial and fungicidal activities even than polymeric chitosan [28,29,72]. It is noteworthy that chitinases, lysozyme, and various non-specific hydrolases are studied much more often than chitosanases for preparation of LMWC [21,32,35,71,73]. In this work, the comparative analysis of partial destruction of chitosan DD 85% by the purified chitinase 73 kDa and chitosanase 40 kDa from Bt-387 indicated the possibility to regulate the degree of substrate hydrolysis in terms of molecular weight, solubility and antimicrobial activity of final product (Figure 1). The findings evidence on the principal promise of both chitosanase and chitinase applications for the production of the soluble COS with DP = 5–10 and LMWC manifesting antifungal effects. In terms of hydrolysis time and molecular-mass characteristics of the produced LMWCs, the efficiency of chitinase Bt-387 is comparable to that of reported chitinases and non-specific hydrolases from diverse origins [21,35,75], while chitosanase Bt-387 has undeniable advantage. The further optimization of the crucial parameters for the hydrolysis of chitosan (temperature, incubation time, ionic strength, pH, the substrate, and enzyme concentrations) using this enzyme could would allow to develop a model for controlled production of targeted COS and LMWC, similar with the one proposed before for the preparation of COSs with specific DP and DD values [22].

The higher oligomers of chitosan (DD 85%), which were produced at both limited (1 h) and more extensive (4 h) hydrolysis, contained exclusively GlcN residues on its reducing end, according to ^13^C-NMR spectra analysis (Figure 9a,b). It indicates that chitosanase Bt-B387 can catalyze the hydrolysis of only two types of glycosidic bonds—the main GlcN–GlcN and mixed GlcN–GlcNAc. That is, it belongs to class III chitosanases according to the classification based on reaction specificity [76]. The specific action of the chitinase B-387 during depolymerization of chitosan (DD 85%) was manifested by the presence of (α + β)-anomeric signals in the ^13^C-NMR spectrum of the resulting oligomers (M_w_ ~15.4 kDa), corresponding to both (GlcN) and (GlcNAc) residues. At the same time, the ratio of terminal GlcN/GlcNAc was about 2.8:1, which is associated with the predominance of deacetylated groups in the original polymer and may also be associated with the ability of the chitinase to hydrolyze additionally both GlcNAc–GlcN and GlcN–GlcNAc bonds (Figure 8b). The last fact supports the LC/MS-analysis data (Table 4) and explains the higher activity of the chitinase toward partially N-acetylated chitosan (DD 50%) compared to colloidal chitin. This model contradicts the current view of the specificity of chitinases to mixed residues, indicating that chitinases of GH18 family (dominating in most bacteria) are able to hydrolyze only GlcNAc–GlcN type of linkage, in the contrast to the rarer chitinases GH19 hydrolyzing exclusively GlcN–GlcNAc [77]. Nevertheless, chitinase B-387 obviously exerts the less selectivity against mixed types of linkages compared to chitosanase, which can be promising for depolymerization strategies based on preparation of bioactive COSs from partially acetylated chitosan.

As mentioned above, chitosanase B-387 demonstrated better efficiency than chitinase in antifungal activity of the produced COS according to the evaluation of ED_50_ for in vitro inhibition of *B. sorokiniana* (Table 5). However, it is a relative index that varies often depending on the taxonomic position of tested culture and an antifungal test method [28,29,30,35,78,79,80,81,82,83]. Nevertheless, similar tendency was observed for the growth-inhibiting activity of the chitosan oligomers against other species of plant pathogenic fungi in terms of its MIC values. Regarding all else, the main findings were in agreement with the common concept of a gradual decrease in COS fungicidal activity in the course of chitosan enzymatic hydrolysis from higher to lower oligomeric products [28,29,79,84,85,86]. For example, the LMWC fractions produced by both chitosanase and chitinase during limited time of hydrolysis (1–2 h) demonstrated obviously higher fungal growth inhibition activity compared to lower oligomers generated by the same enzymes during long-term (4 and 24 h) hydrolysis processes (Table 6). It is possible that the slighter antifungal effect of the chitosan oligomers against the strains of *A. alternata* and *B. sorokiniana* compared to *Fusarium* species may be associated with their synthesis of melanin-like pigments, which play a protective role against the influence of physicochemical and biotic environmental factors. The high melanin content in cell wall of hyphae and spores of *A. alternata* and *B. sorokiniana* significantly increases their hydrophobicity, which can prevent the effective interaction of chitosan oligomers with their cellular structures. This assumption is indirectly supported by data demonstrating enhancing of chitosan fungicidal activity against the *Aspergillus flavus* and *A. parasiticus* when its hydrophobic properties are increased by chemical modification [87]. The lytic action of the relatively large and smaller chitosan oligomeric molecules towards several microorganisms is commonly known and obviously determined by their interaction ability with negatively charged cytoplasmic membrane and cell wall of bacteria and fungi [29,83,86,88,89,90]. More powerful and destructive influence of the LWMC with Mw 45.3 kDa against *Fusarium* species apparently may be due to its thinner cell wall structures and stronger negative charge of the cell membrane compared to the melanin-enriched and highly hydrophobic hyphal cells of *Alternaria* and *Bipolaris* strains. The absence of a visible effect from lower oligomers with M_w_ 2.4 kDa on fungal morphology can probably be explained by other, mainly intracellular, priority targets of its action [89,91]. The exact mechanism of the antifungal action exerted by COS still remains undisclosed completely due to varied sensitivity even between tested strains of same species, heterogeneity of COS mixtures, and significant dependence of their activity on DD, DP and other parameters. The main proof of action of COS is its binding to fungal plasma membrane phospholipids and cell wall constituents including proteins, glycoproteins, etc. [28,29]. The binding ability of positively charged COS directly correlates with the negative charge intensity of plasma membrane in fungal cells [86]. These interactions resulted in alteration of cell membrane permeability, K^+^-efflux, intracellular acidification, increased transmembrane potential. and Ca^2+^ uptake [86]. Large concentrations of COS cause total disruption of the plasma membrane and rapid cell death. Unlike chitosan, COS can penetrate fungal cells, interact with DNA and other negatively charged macromolecules, blocking the transcription processes, and interfering with the expression of essential proteins and enzymes [28,83].

Thus, the comparative antifungal assay of enzymatic chitosan hydrolysates evidences that chitosanase B-387 generates oligomers with more pronounced growth-inhibiting effects than chitinase from the same strain. This trend was observed both in higher and lower COS prepared by partial or extensive hydrolysis of the polysaccharide, respectively. Although there is an opinion that chitosanases are less controllable enzymes for the production of antimicrobial COSs due to rapid hydrolysis of chitosan (DD 85%) to inactive and weakly active chitobiose, chitotriose, and chitotetraose, the findings of this study demonstrate the functional flexibility of chitosanase B-387 for managed depolymerization of chitosan allowing to produce a wide range of COSs exerting substantial antifungal activity. In turn, chitinase B-387 is also suitable for controllable chitosan hydrolysis, but its effectiveness is limited by substrate DD and manifests in lower solubility and antifungal activity of the resulting oligomers.

## 5. Conclusions

The findings of the research clearly illustrate the different potentials of chitinase(s) and chitosanase from *B. thuringiensis* B-387 for the production of antifungal chitosan oligomers. In the biotechnological aspect, chitosanase (40 kDa) has essential advantages over chitinase due to its 10–20-fold higher and more stable production in the culture without need for an expensive inducer substrate, the synthesis of a single enzyme isoform, as well as simpler and more effective purification protocol for homogenous enzyme preparation. This enzyme may be easily obtained almost without the chitinase admixtures through the batch culture in LB or other nutrient media comprising the sources of organic nitrogen. As a distinct from chitosanase, *B. thuringiensis* B-387 produces chitinase as an enzyme complex, including, at least, three proteins with Mw 73, 60, and 36 kDa. The physicochemical characteristics of chitinase 73 kDa and chitosanase 40 kDa, such as pH and temperature optima and stabilities, are very close, but both enzymes differ significantly in their kinetic properties and substrate specificity. Apart from the obvious manifold higher rate of hydrolysis of chitosan DD 85%, chitosanase demonstrates stricter specificity to mixed bonds compared to the chitinase. It splits only GlcN–GlcNAc in the pattern of class III chitosanases, while chitinase well hydrolyzes both types of mixed bonds that manifests in a sharp increase in its hydrolytic activity towards partially N-acetylated chitosan (DD 50%). This fact accents the advantages of chitinase in the production of partially acetylated COS displaying diverse bioactivities. At deep hydrolysis of chitosan DD 85%, chitinase may also have a better potential for generating longer-chain COS (PD = 5–7) as dominant final products with higher antimicrobial activity compared to products (n = 2–4) released by chitosanase. However, under limited polymer hydrolysis, chitosanase produces a mixture of bioactive COS with similar sizes (Mw 2.4 kDa) and significantly higher final yields. Moreover, oligomers produced by chitosanase both at partial and deeper hydrolyses of chitosan (DD 85%) demonstrate generally better functionality in terms of solubility and antifungal activity. Thus, within a simple strategy for antifungal COS production, chitosanase B-387 is a more controllable and promising enzyme than chitinase(s). Nevertheless, further development and diversification of functional COS production may favor for primary application of chitinase or its concerted action together with chitosanase.

## Figures and Tables

**Table 1:** Dynamics of bacterial growth, extracellular protein, and protease production, as well as pH changes in cultivation media of *B. thuringiensis* B-387 (96 h, 36.5 °C 220 rpm).

Cultivation Period	LB Broth				
	Growth Characteristics		Protein in CS ^a^, mg/mL	Protease, AU ^b^/mL	pH of CS
	OD_600_	×10^7^ CFU/mL			
0 h	0.05 ± 0.01	1.77 ± 0.76 ^c^	–	0	6.81
24 h	6.01 ± 0.14	66 ± 8	6.87 ± 0.35	0.093 ± 0.010	8.01
48 h	5.09 ± 0.30	14.8 ± 3	8.87 ± 0.86	0.587 ± 0.057	8.70
72 h	3.16 ± 0.10	3.8 ± 0.60	8.93 ± 0.75	0.394 ± 0.029	8.92
96 h	2.47 ± 0.10	0.02 ± 0.004	9.38 ± 0.60	0.115 ± 0.007	9.02
	The medium with 1% (*w*/*v*) of colloidal chitin (CCM)				
0 h	– ^d^	1.77 ± 0.76 ^c^	–	0	6.53
24 h	–	56 ± 15	5.66 ± 0.37	0.139 ± 0.012	5.90
48 h	–	12 ± 2	5.35 ± 0.45	0.165 ± 0.014	8.30
72 h	–	0.10 ± 0.02	3.73 ± 0.42	0.078 ± 0.008	8.80
96 h	–	0.05 ± 0.001	3.85 ± 0.38	0.089 ± 0.014	8.99

^a^ CS—culture supernatant; ^b^ AU—arbitrary units (see Materials and Methods); ^c^ Initial titer value of bacterial cells inoculated into media; ^d^ Absorbance evaluation of the bacterial growth in CCM was not carried out by reason of high turbidity of colloidal chitin hindering to adequate biomass measurement especially in first days of cultivation.

**Table 2:** Purification of chitosanase (1) and chitinase (2) from *B. thuringiensis* B-387.

Step of Purification	Total Enzyme Activity, U		Total Protein, mg		Specific Activity, U/mg		Purification Degree, -Fold		Enzyme Yield, %	
	1	2	1	2	1	2	1	2	1	2
CF ^a^, (96 h)	1619.68	119.25	4722.3	1040	0.343	0.064	1	1	100	100
VivaFlow 200 module (30 kDa)	931.32	107.46	822.72	870.96	1.132	0.123	3.3	1.9	58	90
Affinity adsorption ^b^	730.48	69.20	204.79	441.94	3.567	0.157	10.4	2.5	45	58
Phenyl-Sepharose CL 4B	—	34.79	—	22.79	—	1.527	—	24	—	29
CM-Sepharose Fast Flow	147.39	—	4.57	—	32.252	—	94	—	9	—

^a^ CS—culture supernatant; ^b^ affinity adsorption step was performed on 0.5% colloidal chitosan and 1% colloidal chitin for chitosanase and chitinase, respectively.

**Table 3:** The main biochemical and catalytic properties of extracellular chitosanase and chitinase produced by *B. thuringiensis* B-387.

Characteristics	Chitosanase	Chitinase
Temperature optimum, °C	55	55
Thermal stability, °C ^a^	60	60
Optimal pH	6.5	6.5
pH stability ^b^	6–10	4–9
pI	7.5–8	ND
Molecular weight	40	73
K_M_, mg/mL (toward chitosan DD 85%)	0.22	>5.4
V_max_ (toward chitosan DD 85%), μM × mL^−1^ × mg^−1^	56.52	0.124
V_max_ (toward colloidal chitin), μM × mL^−1^ × mg^−1^	ND ^c^	14.1
Action mechanism to a specific substrate	Endo- ^d^	Exo- ^e^
Final products of chitosan (DD 85%) hydrolysis	(GlcN)_2-4_ ^f^	(GlcN) _2-4_ ^f^
Major products of colloidal chitin hydrolysis	NA ^g^	(GlcNAc)_2_ and GlcNAc ^d^
Catalytic activity, nkat (chitosan DD 85%)	942.19	ND ^a^
k_cat_, s^−1^	5.84 × 10^3^	ND ^a^
Inhibition by metal cations and detergents	10 mM Zn^2+^, Hg^+^, Cd^2+^, Ag^+^ Fe^+2^ and 10 mM SDS	1–10 mM Hg^+^, Cd^2+^, Ag^+^, Fe^+2^, and 10 mM SDS
Activation by metal cations and surfactants	10 mM tween-80	10 mM tween-80

^a^ Thermal stability was defined as maintaining 100% of original activity after a 1 h pre-incubation at 60 °C; ^b^ pH stability was defined as maintaining 100% of original activity after a 24 h pre-incubation in the indicated pH range at 5 °C; ^c^ ND—no data available; ^d^ toward chitosan DD 85%; ^e^ toward colloidal chitin; ^f^ according to the TLC data; ^g^ NA—no activity.

**Table 4:** The list of the main oligomeric products generated through deep hydrolysis of chitosan DD 85% and DD 50% by the purified enzymes from *B. thuringiensis* B-387 according to LC-MS analysis (the data of chitosan DD 50% hydrolysis products are shown only for chitinase 73 kDa).

No.	Main Ion Peaks, *m*/*z* (Proposed Adducts and Charges)	COS Product	Retention Time, min	Rel. Content ^a^, %
Chitosan DD 85% degradation products by chitosanase 40 kDa ^b^				
1	363.14 (Na^+^), 341.14^+^, 342.17 (H^+^)	(GlcN)_2_	5.47	30
2	502.23, 503.23 (H^+^), 524.2 (Na^+^)	(GlcN)_3_	6.29	54.2
3	332.15^2+^, 332.65^2+^, 333.15 (2H^2+^) 663.29^+^, 663.79^+^, 664.29 (H^+^)	(GlcN)_4_	7.30	15
4	412.69^2+^	(GlcN)_5_	7.30	0.1
5	433.69^2+^, 705.3^+^, 705.8^+^	GlcNAc-(GlcN)_4_	7.30	0.7
Chitosan DD 85% degradation products by chitinase 73 kDa ^c^				
1	341.15^+^, 342.15 (H^+^), 363.13 (Na^+^)	(GlcN)_2_	5.65	12.2
2	502.22^+^, 503.22 (H^+^), 524.2 (Na^+^)	(GlcN)_3_	5.70	12.1
3	544.2+, 545.22 (H^+^), 566.21(Na+)	(GlcN)_2_-GlcNAc	5.70	1.6
4	663.29^+^, 663.79^+^, 664.28 (H^+^), 663.31^+^, 332.14^2+^, 332.65^2+^, 333.15 (H^+^)	(GlcN)_4_	6.80	7
5	412.68^2+^, 413.18^2+^, 413.68 (2H^2+^), 824.41^+^, 824.37^+^	(GlcN)_5_	7.81	19.3
6	433.68^2+^, 435.18 (2H^2+^)	(GlcN)_4_-GlcNAc	7.81	2.7
7	493.21^2+^, 493.71^2+^, 494.21 (2H^2+^), 493.23^2+^, 493.73^2+^, 494.23 (2H^2+^)	(GlcN)_6_	7.98	19.2
8	514.22^2+^, 514.72^2+^, 515.21 (2H^2+^), 514.24^2+^, 514.74^2+^	GlcNAc-(GlcN)_5_	8.00	6
9	573.77^2+^, 574.27^2+^, 574.77 (2H^2+^)	(GlcN)_7_	8.68	13.6
10	594.78^2+^	(GlcN)_6_-GlcNAc	10.01	0.4
11	654.30^2+^, 654.80^2+^, 655.31 (2H^2+^)	(GlcN)_8_	10.05	3.4
12	755.85^2+^	(GlcN)_8_-GlcNAc	10.57	0.1
13	734.83^2+^, 735.33^2+^	(GlcN)_9_	11.16	0.2
Chitosan DD 50% degradation products by chitinase 73 kDa ^d^				
1	222.10^+^, 222.23^+^, 222.32^+^, 222.39^+^, 222.55^+^, 223.10 (H^+^)	GlcNAc	4.11	12
2	204.09 (Na^+^)	GlcN	4.09	7.7
3	343.18 (2H^2+^), 341.16^+^, 363.14 (Na^+^)	(GlcN)_2_	4.30	5.4
4	443.2^+^, 444.19 (H^+^), 447.17 (2H^2+^)	(GlcNAc)_2_	4.35	4.8
5	405.15 (Na^+^), 383.17^+^, 383.35^+^, 383.55^+^, 383.76^+^, 384.17 (H^+^), 406.16 (H^+^ Na^+^), 405.15 (Na^+^)	GlcN-GlcNAc	4.92	22.7
6	502.23^+^, 503.24 (H^+^), 524.22 (Na^+^)	(GlcN)_3_	6.24	8.4
7	544.24^+^, 545.24 (H^+^), 566.23 (Na^+^)	(GlcN)_2_-GlcNAc	6.25	12.9
8	608.22^+^	GlcNAc-GlcN-GlcNAc (presumably)	6.25	0.7
9	663.27^+^	(GlcN)_4_	7.16	0.8
10	705.28^+^, 706.28 (H^+^)	(GlcN)_3_-GlcNAc	7.17	0.8
11	747.30^+^, 748.27 (H^+^)	GlcNAc-(GlcN)_2_-GlcNAc	7.20	0.8
12	433.69^2+^, 434.20 (2H^2+^), 434.7 (2H^2+^)	(GlcN)_4_-GlcNAc	7.67	22.5
13	412.69^2+^	(GlcN)_5_	7.92	0.4

^a^ The relative content was calculated based on the sum of absolute intensities of the signals relating to all clearly identified COS ions presented for every hydrolysate mixture taken as 100%. The most abundant products are highlighted in bold. ^b^ Purified chitosanase (11 mU) diluted in deionized water (pH 6) (~57 mU/mL) was added to equal volume of 0.5% (*w*/*v*) chitosan DD 85% solution (pH 6) and incubated for 5 h at 50 °C. ^c, d^ Purified chitinase (0.46 U) diluted in deionized water (~2.32 mU/mL) was added to equal volume of 0.5% (*w*/*v*) chitosan DD 85% or DD 50% solutions (pH 6) and incubated for 5–6 h at 50 °C.

**Table 5:** Molecular weight characteristics, solubility, yield, and fungicidal activity of oligomers prepared through hydrolysis of chitosan (DD 85%) by purified chitosanase and chitinase of *B. thuringiensis* B-387.

Incubation Time and Temperature	Fraction	M_n_, ^a^ kDa	M_w_, ^b^ kDa	PDI ^c^	Solubility ^d^, g/L	Yield, %	Inhibition of *B. sorokiniana*, ED_50_, mg/mL ^e^
–	Initial polymeric chitosan	128.5	369.2	2.87	–	–	0.10
Depolymerization by chitosanase (~2.5–11 U/g substrate)							
1 h at 50 °C	The precipitated fraction after adding 0.5 M NaOH	22.5	66.5	2.96	48.1	48.1	0.29
2 h at 50 °C		23.25	45.34	1.95	57.7	29.8	0.45
2 h at 50 °C	Supernatant after adding 0.5 M NaOH	1.96	2.19	1.12	63.0	8	2.50
4 h at 50 °C	Total hydrolysate	1.92	2.41	1.26	64.9	68	1.29
Depolymerization by chitinase (~8–14 U/g substrate)							
1 h at 50 °C	The precipitated fraction after adding 0.5 M NaOH	27.43	71.74	2.62	24.4	43.4	0.69
1 h at 50 °C	Supernatant after adding 0.5 M NaOH	2.52	2.89	1.15	42.8	<6	3.00
24 h at 37 °C ^f^	Total hydrolysate	7.32	15.38	2.10	21.6	73	1.22

^a^ M_n_—number-average molecular weight; ^b^ M_w_—weight-average molecular weight; ^c^ PDI—polydispersity index (M_w_/M_n_); ^d^ In deionized water; ^e^ ED_50—_effective concentration of LMWC resulting in 50% fungal growth inhibition in PDB; ^f^ Although the chitinase is relatively thermostable at 50 °C, its long-term incubation (24 h) at this temperature could result to significant reduction in its activity, consequently, it was incubated at milder temperature (37 °C) for better efficiency of the hydrolysis process.

**Table 6:** The minimum inhibitory concentration (MIC) values of various fractions of oligomers generated at hydrolysis of chitosan (DD 85%) by purified chitosanase and chitinase of *B. thuringiensis* B-387 against several strains of phytopathogenic micromycetes.

Tested Fungal Strain	MIC, μg × mL^−1^				
	Chitosanase Products/Hydrolysis Time			Chitinase Products/Hydrolysis Time	
	2 h		4 h	1 h	24 h
	45.3 kDa ^a^	2.19 kDa ^b^	2.4 kDa ^c^	71.7 kDa ^a^	15.4 kDa ^c^
*A. alternata* VKM F-3047	200 ± 15	2250 ± 250	750 ± 55	310 ± 30	575 ± 50
*B. sorokiniana* IB G-12	240 ± 20	1850 ± 200	675 ± 45	320 ± 35	590 ± 55
*F. culmorum* VKM F-844	<21	1520 ± 140	230 ± 20	38 ± 5	200 ± 20
*F. gibbosum* VKM F-848	<19	NI ^d^	665 ± 55	55 ± 5	540 ± 45
*F. graminearum* VKM F-1668	<20	NI	485 ± 35	90 ± 10	390 ± 35
*F. oxysporum* VKM F-137	<21	2500 ± 200	150 ± 20	75 ± 5	145 ± 10
*F. solani* VKM F-142	<20	2500 ± 170	295 ± 25	65 ± 10	280 ± 30

^a^ NaOH-precipitated fractions; ^b^ NaOH-unprecipitated (supernatant) fraction; ^c^ total hydrolysate fractions; ^d^ at tested concentrations ≤ 2500 μg × mL^−1^; NI—no growth inhibition.

## Data Availability

The original contributions presented in this study are included in the article. Further inquiries can be directed to the corresponding author.

## Supplementary Materials

The following supporting information can be downloaded at: https://www.mdpi.com/article/10.3390/biotech14020035/s1, Figure S1: The diagram illustrating the main isolation and purification stages of the chitinase and chitosanase from the culture supernatant of *B. thuringiensis* B-387; Figure S2: Hydrophobic chromatography of the partially purified chitinase fraction from *B. thuringiensis* B-386 on the column (2.5 × 10 cm) packed with Phenyl-Sepharose CL 4B; Figure S3: The comparative production of chitinase and chitosanase by the strain *B. thuringiensis* B-387 in standard LB broth and LB broth supplemented with colloidal chitin; Figure S4: Bacterial growth rates and changes in its protease production and pH change during submerged cultivation of *B. thuringiensis* B-387 in LB broth; Figure S5: SDS-PAGE and zymographic assay of crude and purified chitinase(s) secreted by *B. thuringiensis* B-387; Figure S6: The hypothetical scheme of the chitosan (DD 50%) hydrolysis by the chitinase from B. thuringiensis B-387 in terms of the probable polymer acetylation pattern. Figure S7: Thin-layer chromatography of oligomers generated at partial and extensive hydrolysis of chitosan DD 85% by the purified chitosanase of *B. thuringiensis* B-387 on Silica gel 60 F_254_ sheets; Table S1: The substrate specificity of the purified chitinase and chitosanase from *B. thuringiensis* B-387.
