# Supplementary material for: Comparative Potential of Chitinase and Chitosanase from the Strain Bacillus thuringiensis B-387 for the Production of Antifungal Chitosan Oligomers
Source: BioTech (Basel). 2025 May 8;14(2):35. doi: 10.3390/biotech14020035 (PMC12101196; doi:10.3390/biotech14020035)
Supplement: Supplementary file 1 [file biotech-14-00035-s001.zip › biotech-3581299-supplementary.pdf]

BioTech

Comparative potential of the chitinase and chitosanase from the strain *Bacillus thuringiensis* B-387 for production of antifungal chitosan oligomers

Gleb Aktuganov[a1*], Alexander Lobov[b], Nailya Galimzianova[a], Elena Gilvanova[a], Lyudmila Kuzmina[a], Polina Milman[a], Alena Ryabova[a], Alexander Melentiev[a], Sergey Chetverikov[a], Segey Starikov[a], Sergey Lopatin[d]

[a] *Institute of Biology, Ufa Federal Research Center of Russian Academy of Sciences, 69, Prospect Oktyabrya, 450054 Ufa, Russia*
[b] *Institute of Organic Chemistry, Ufa Federal Research Center of Russian Academy of Sciences, 71, Prospect Oktyabrya, 450054 Ufa, Russia*
[c] *Institute of Bioengineering of Federal Research Center "Fundamentals of Biotechnology" of Russian Academy of Sciences, 7, bld. 1, 60 let Oktyabrya prospect, 117312 Moscow, Russia*

[1] *Corresponding author. Tel.: +7 347- 2355362; fax: +7 347 2356247.
E-mail address: gleakt@anrb.ru (G.E. Aktuganov)

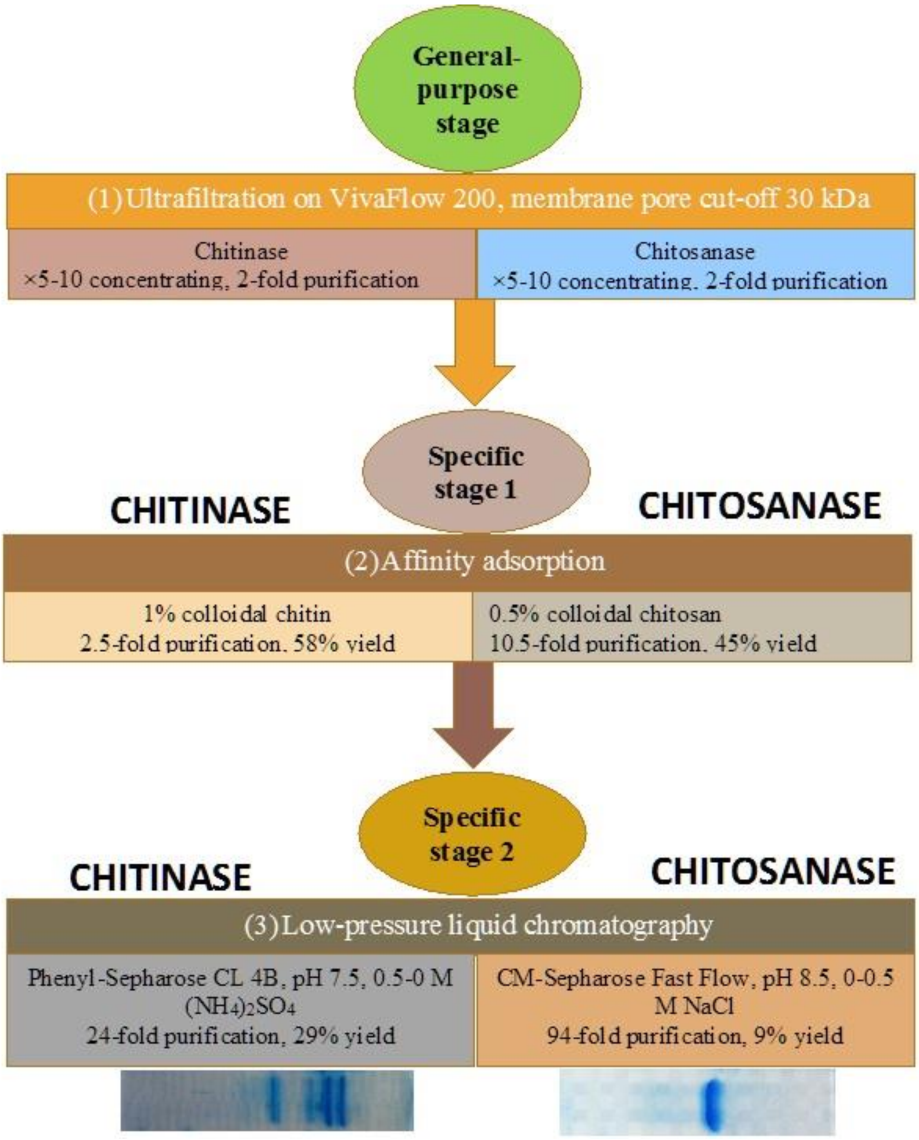

Figure S1. The diagram illustrating the main isolation and purification stages of the chitinase and chitosanase from the culture supernatant of *B. thuringiensis* B-387.

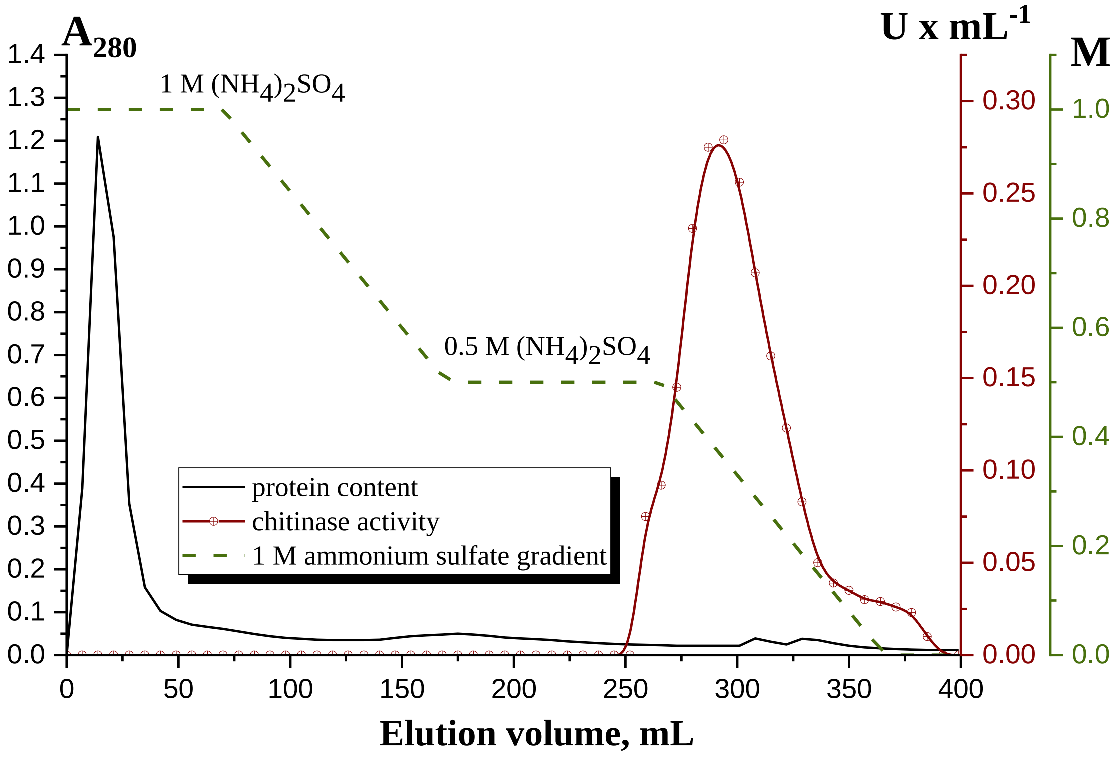

Figure S2. Hydrophobic chromatography of the partially purified chitinase fraction (after ultrafiltration and affinity sorption steps) from *B. thuringiensis* B-386 on the column (2.5×10 cm) packed with Phenyl-Sepharose CL 4B. The main eluent is Tris-HCl (25 mM, pH 7.5); flowrate 1.5 mL/min, fractions volume 7 mL.

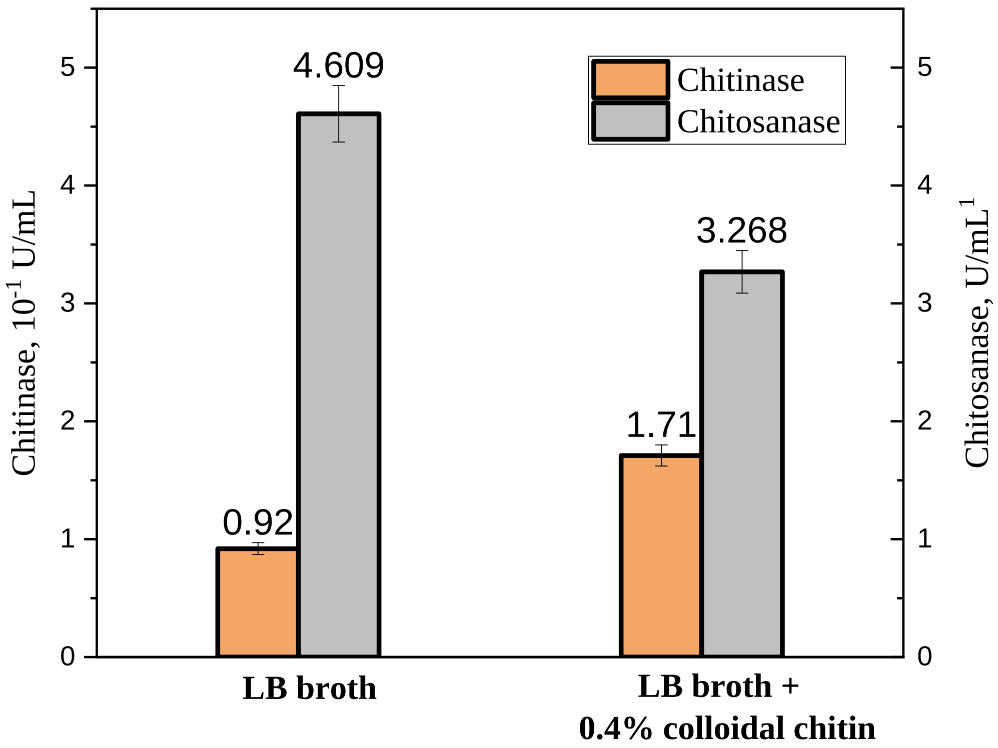

Figure S3. The comparative production of chitinase and chitosanase by the strain *B. thuringiensis* B-387 in standard LB broth and LB broth supplemented with colloidal chitin (3 days of cultivation at 36.5°C and 220 rpm).

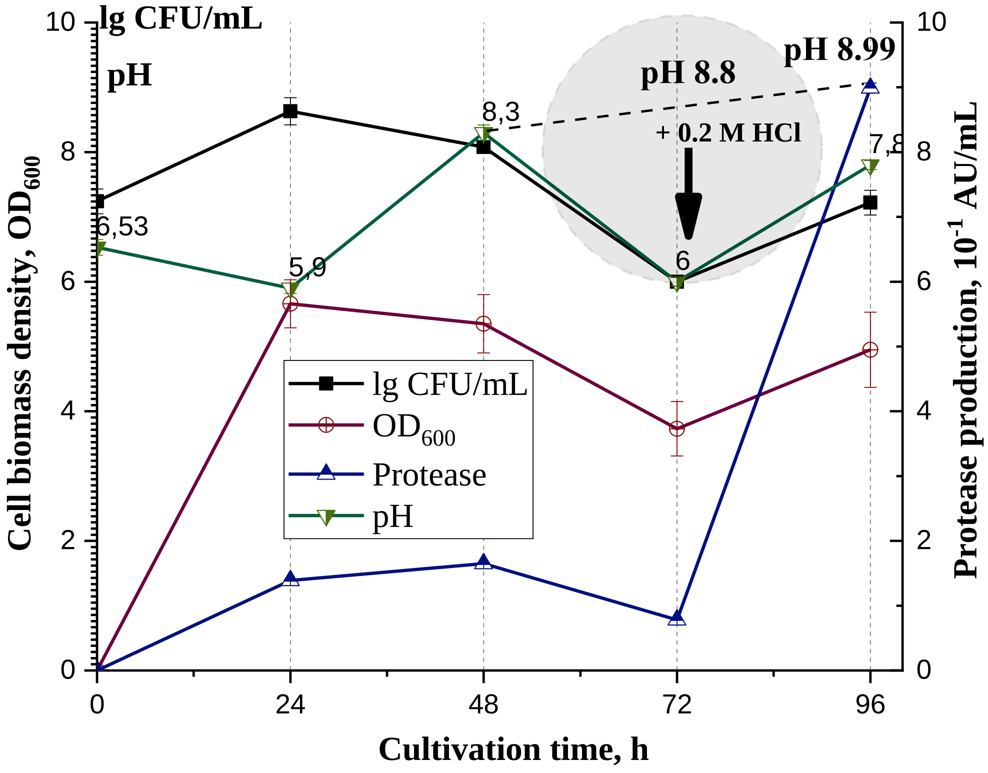

Figure S4. The dynamics of *B. thuringiensis* B-387 biomass growth and cell titer, protease production and the culture filtrate's pH change during its cultivation in LB broth (36°C, 220 rpm). The arrow within grey circle indicates the time point of pH adjustment in culture broth from 8.8 to 6 through acidification resulting to renewal of bacterial growth and steep increase of protease activity. The dashed line indicates continued dynamics of pH change in period between 48 and 96 h without medium acidification.

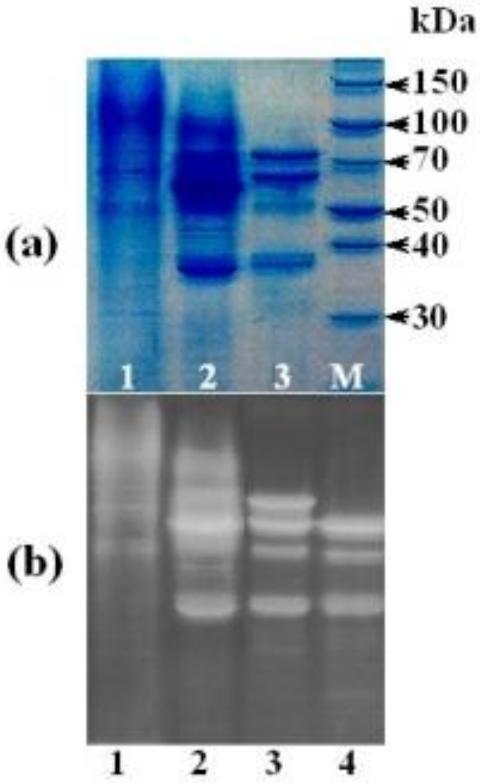

Figure S5. SDS-PAGE (a) and zymographic assay (b, negative) of crude and purified preparations of the chitinase(s) secreted by *B. thuringiensis* B-387 during growth in CCM medium. The numbers 1, 2 and 3 are identical for (a) and (b) images corresponding to the culture supernatant, the fraction adsorbed by 0.5% colloidal chitin and the fraction purified using hydrophobic chromatography, respectively. The number 4 in image (b) denotes the residual chitinase fraction non-adsorbed on colloidal chitin after additional purification on Phenyl-Sepharose CL 4B. M- molecular weight standard markers PageRuler Broad Range. The zymographic results are depicted as negative image of 7.5% polyacrylamide gel replica containing 0.05% (w/v) glycol-chitin and photographed in UV-light after 3-h pre-incubation (37°C) and staining with Calcofluor White M2R dye.

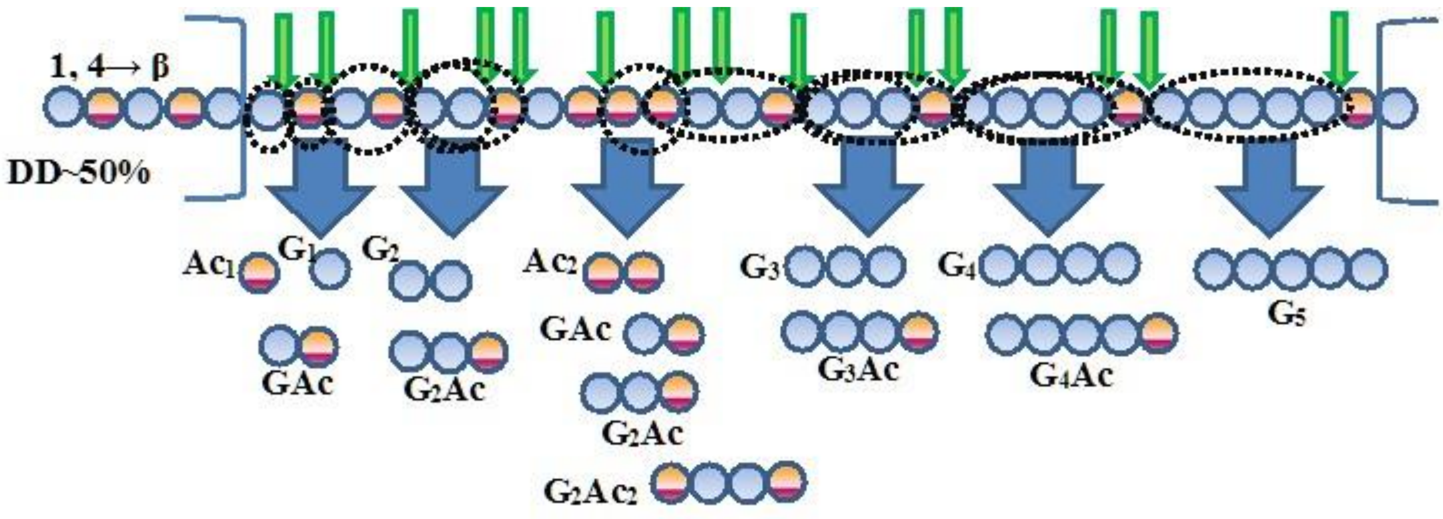

Figure S6. The hypothetical scheme of the chitosan (DD 50%) hydrolysis by the chitinase from B. thuringiensis B-387 in terms of the probable polymer acetylation pattern. The generated monomers and oligomers: Ac_1 – GlcNAc; Ac_2 – $GlcNAc_2$; $G_{2\text{-}5}$ – oligomers of GlcN with n=2-5; G_nAc – oligomers of GlcN containing a single GlcNAc residue; G_2Ac_2 – the sole detected tetramer containing by couples of GlcN and GlcNAc residues. The small green arrows indicate the hydrolyzed bonds; the roundish zones with dashed lines indicated the cleaved fragments in the chitosan molecule. The bluish circles correspond to GlcN residues, and two-colored circles – to GlcNAc residues.

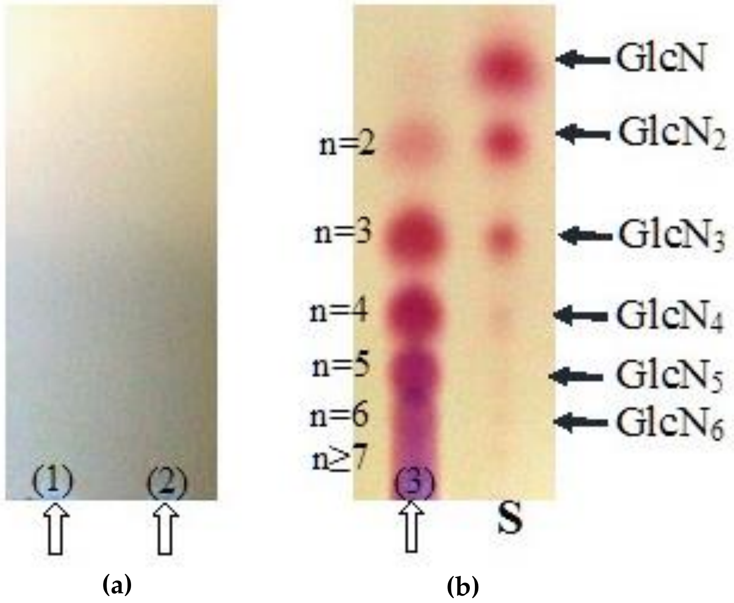

Figure S7. Thin-layer chromatography of oligomers generated at partial (A) and extensive (B) hydrolysis of chitosan DD 85% by the purified chitosanase of *B. thuringiensis* B-387 on Silica gel 60 F_{254} sheets (10×20 cm). (1), (2) and (3) – the products generated after 1, 2 (enzyme/substrate~2.6 U/g) and 4 h (enzyme/substrate~10.7 U/g) of incubation at 50°C, respectively. The wide hollow arrows from the bottom indicate the application points for analyzed products. S – commercial standards including glucosamine (GlcN) and chitosan oligomers with DP 2-6. N – DPs values of the resultant short-chain COSs.

Table S1. The substrate specificity of the purified chitinase and chitosanase from *B. thuringiensis* B-387

**The substrate, 0,5% (w/v)**	**The relative activity, %**	
	Chitinase 73 kDa	***Chitosanase 40 kDa*** [a]
Colloidal chitin	100	0
Soluble chitosan DD 85%	18.4	100
Soluble chitosan DD 50%	222.6	29.2
Laminarin	0.9	0
β-glucan	0.5	0
CM-cellulose	0.9	0
Amorphic cellulose	0.5	0
Xylan	0	0
Galactomannan	0	0

[a] The data were taken from [40].
